# ELAS1 induces apoptotic death in adenocarcinoma DU145 and squamous-cell carcinoma SAS cancer cells, but not in normal KD cells

**DOI:** 10.18632/oncotarget.20696

**Published:** 2017-08-24

**Authors:** Toshihiro Uchihashi, Kaori Ota, Yusuke Yabuno, Shouichi Ohno, Kohshiro Fukushima, Yoko Naito, Mikihiko Kogo, Norikazu Yabuta, Hiroshi Nojima

**Affiliations:** ^1^ First Department of Oral and Maxillofacial Surgery, Graduate School of Dentistry, Osaka University, Osaka 565-0871, Japan; ^2^ Department of Molecular Genetics, Research Institute for Microbial Diseases, Osaka University, Osaka 565-0871, Japan

**Keywords:** cyclin G1, p53, DU145, SAS, adenovirus

## Abstract

We previously reported that an ELAS1 peptide containing 29 amino acids induces apoptotic death in U2OS human osteosarcoma cells following DNA double-strand break insults. Here, we show that ELAS1 also caused apoptosis in prostate adenocarcinoma DU145 cells and tongue squamous-cell carcinoma SAS cells. ELAS1 appears to be safe because it induced apoptosis only in cancer cells, not in normal KD cells. Because the effect of ELAS1 is dependent on increased stability of p53 and enhanced phosphorylation of p53-S46, we exogenously expressed wild-type p53 protein to fully promote ELAS1-mediated induction of apoptosis in SAS cells. Interestingly, simultaneous expression of Myc-ELAS1 and FLAG-p53 mediated by an internal ribosome entry site efficiently induced apoptosis in SAS cells. Moreover, we prepared a recombinant adenovirus that simultaneously expressed Myc-ELAS1 and FLAG-p53. This adenovirus also killed SAS cells, as determined by a cell viability assay, in the presence of camptothecin, an inducer of DNA double-strand breaks. Moreover, nude mice harboring Myc-ELAS1-expressing SAS cells lived longer than mice harboring Myc-vector-expressing SAS cells, suggesting the usefulness of ELAS1 *in vivo*. Notably, Cy5-tagged ELAS1-t, which contained only ten amino acids, also efficiently induced apoptosis in both DU145 and SAS cells, suggesting the usefulness of ELAS1-t as a peptide. Taken together, our results suggest that ELAS1 is therapeutically useful as a peptide drug.

## INTRODUCTION

Radiation therapy (RT) using concentrated beams of ionizing radiation (IR) is curative in various cancers [1]. IR selectively kills cancer cells because it induces DNA lesions, including DNA double-strand breaks (DSBs). However, because a high dose of IR is also toxic to normal cells, RT is considered to be a once-in-a-lifetime treatment [2]. Thus, an adjuvant that helps to kill cancer cells with a much lower dose of IR and the same therapeutic effectiveness has been sought [3]. Some types of chemotherapeutic drugs, such as camptothecin (CPT) and irinotecan, also induce DSBs in cancer cells [4, 5]. CPT causes the persistence of single-strand DNA breaks by stabilizing topoisomerase I-DNA cleavage complexes [6]. CPT is a natural product isolated from a Chinese tree (*Camptotheca acuminate*) and has been used as a Chinese herbal medicine to treat cancer [7]. Because CPT has severe side effects, numerous CPT analogues such as irinotecan (Camptosar™) have been synthesized and are presently used in cancer chemotherapy [8].

We recently reported that a peptide containing 29 amino acids (aa) named ELAS1 [i:laz wΛn] would serve as such an agent because apoptosis of Myc-ELAS1-expressing human osteosarcoma (U2OS) cells is induced by one-tenth of the effective dosage of γ-irradiation in Myc-vector-expressing cells [9]. Moreover, apoptosis of Myc-ELAS1-expressing U2OS cells is efficiently induced by only one-hundredth (CPT) or one-fifth (irinotecan) of the amounts of drugs required to induce this effect in Myc-vector-expressing cells [9]. ELAS1, a peptide corresponding to the association domain of Cyclin G1 (CycG1) with protein phosphatase 2A (PP2A), associates with the B'γ subunit of PP2A [10, 11] and competitively inhibits the association with CycG1 [12, 13]. ELAS1 prevents the proper action of the PP2A holoenzyme on its dephosphorylating target p53-pS46, which is activated following DSB insults [9, 14, 15]. Apoptosis is not induced by ELAS2, a 29 aa peptide with a similar aa sequence corresponding to the association domain of Cyclin G2 (CycG2), although ELAS2 also competitively inhibits the association between CycG2 and the B’γ subunit of PP2A [9]. To examine if ELAS1 is effective at the bedside, it must be determined if it also causes efficient apoptosis in other cancer cells in which p53-pS46 remains active because many cancer cells show a high incidence of *TP53* mutations [16–19].

Among common cancers, we selected prostate cancer and tongue cancer cell lines to further study ELAS1 function. DU145 cells harboring the P223L and V274F point mutations but with the wild-type (WT) p53-S46 residue [20] are less sensitive to docetaxel than LNCaP and C4-2 cells, which express functional p53 [21]. Because this phenomenon is due to increased p53-S15 phosphorylation [21], it remains undetermined if ELAS1-mediated apoptosis also occurs in DU145 cells through increased p53-S46 phosphorylation. As a tongue cancer cell line, SAS appears to be suitable to examine the apoptotic function of ELAS1 because it harbors the WT p53-S46 residue, although it has an E336X (X means a stop codon) mutation, generating a truncated p53 protein, according to the mutation list in the TP53 website (http://p53.free.fr/Database/Cancer_cell_lines/p53_cell_lines.html). A large number of *TP53* mutations listed in this website would play a role in personalized medicine by providing targets for drug development and new therapeutic approaches [22].

The aim of this study was to show that ELAS1 is useful as an adjuvant that helps to kill cancer cells with much lower doses of IR, CPT, and irinotecan. To this end, we examined DU145 and SAS cells. Moreover, to develop an efficient method to deliver the ELAS1 peptide into cancer cells, we prepared a recombinant adenovirus that expressed both ELAS1 and WT p53 protein and found that it efficiently killed p53-deficient SAS cells. We also found that ELAS1 could be shortened from 29 aa to ca. 10 aa without loss of its apoptosis-inducing function. These results demonstrate the general usefulness of ELAS1 for use at the bedside in the future.

## RESULTS

### ELAS1 causes apoptosis in DU145 cancer cells

**Figure 1:**
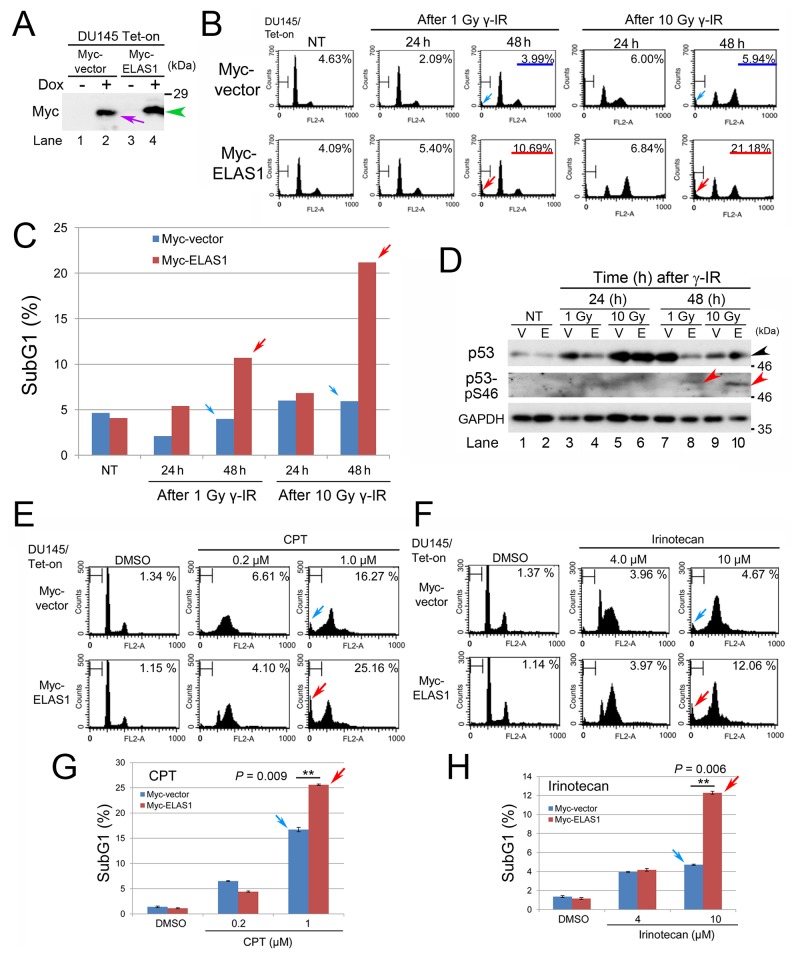
Exogenous expression of ELAS1 causes apoptotic death of DU145 cells after dsDNA insults **(A)** Wb was conducted to show the successful establishment of DU145/Tet-On cells expressing Myc-vector or Myc-ELAS1 in the absence (-) or presence (+) of Dox. The purple arrow and green arrowhead indicate Dox-inducible bands for Myc protein and Myc-ELAS1 protein, respectively. **(B)** FC analysis. DU145/Tet-On cells stably expressing Myc-vector alone or Myc-ELAS1 were treated with 1 or 10 Gy γ-IR for the indicated duration (h) in the presence of Dox. Cells were stained with propidium iodide (PI) and the cell cycle profiles were determined by FC. The percentages correspond to the sub-G1 population of cells. **(C)** The bar graphs show the percentages of subG1 cells as determined by FC. DU145/Tet-On cells expressing Myc-vector or Myc-ELAS1 following Dox-mediated induction were treated with 1 or 10 Gy γ-IR, cultured for 24 or 72 h, and subjected to FC. Data represent the means and standard deviations (SD) of three independent experiments. Red and blue arrows correspond to the subG1 peak of FC data shown in Figure 1B. **(D)** DU145/Tet-On cells expressing Myc-vector (V) or Myc-ELAS1 (E) following Dox-mediated induction were cultured for 24 h and subsequently cultured for the indicated duration (h) in the absence (NT) or presence of 1 or 10 Gy γ-IR treatment. Then, the cell extracts were subjected to Wb with anti-p53, anti-phospho-p53-S46, and anti-GAPDH (loading control) antibodies. The black and red arrowheads indicate the p53 and p53-S46 bands, respectively. **(E, F)** FC analysis of DU145/Tet-On cells expressing Myc-vector or Myc-ELAS1 following Dox-mediated induction in the presence of CPT (E) or irinotecan (F). SubG1 peaks are indicated by blue and red arrows. **(G, H)** The bar graphs show the percentages of subG1 cells as determined by FC. Red and blue arrows correspond to the subG1 peaks of FC data shown in Figure 1E and Figure 1F.

We previously showed that the ELAS1 peptide efficiently causes apoptosis in human osteosarcoma U2OS cells through inhibition of the CycG1-B’γ association, leading to stabilization and activation of p53 [9]. We investigated if this phenotype is applicable to other more prevalent cancers. We first tested human prostate cancer by generating human adenocarcinoma DU145 cells that expressed doxycycline (Dox)-inducible Myc-vector or Myc-ELAS1. Western blot (Wb) analysis confirmed the successful construction of these DU145/Tet-On cells expressing Myc-vector or Myc-ELAS1 in a Dox-dependent manner (Figure 1A). Indeed, Myc-ELAS1 (green arrowhead) migrated slower than Myc-vector alone (purple arrow). Flow cytometry (FC) revealed that Dox-dependent expression of Myc-vector alone and Myc-ELAS1 had no effect on cell cycle progression (column non-treated (NT) in Figure 1B). The subG1 population of Myc-ELAS1-expressing DU145 cells increased to 10.69% and 21.18% at 48 h after exposure to 1 and 10 Gy γ-IR, respectively (red arrows in Figure 1B). By contrast, no change was observed in DU145 cells expressing Myc-vector alone (blue arrows in Figure 1B). Bar graphs of the data clearly show the induction of apoptosis by Myc-ELAS1 (red arrows in Figure 1C) compared with Myc-vector alone (blue arrows in Figure 1C). Wb confirmed that Myc-ELAS1-expressing DU145 cells showed a band corresponding to p53-pS46 (red arrowhead in Figure 1D) at 48 h after treatment with 1 Gy (lane 8) or 10 Gy (lane 10) γ-IR, even when the p53 protein level was not largely increased or rather decreased (black arrowhead in Figure 1D). To examine if the increased subG1 population was actually derived from apoptotic cell death, we conducted the TUNEL assay. Indeed, apoptosis of Myc-ELAS1-expressing DU145 cells was increased at 24 and 48 h after treatment with 1 Gy or 10 Gy γ-IR (Supplementary Figure 1). These results suggest that point mutations (P223L and V274F) of p53 protein do not hamper the ELAS1-mediated apoptosis through phosphorylation of p53-pS46.

To examine if other DNA insults that generate DSBs also caused ELAS1-dependent apoptosis, we added CPT or irinotecan to the culture medium of Myc-vector- or Myc-ELAS1-expressing DU145 cells. Treatment with 1 μM CPT (Figure 1E) or 10 μM irinotecan (Figure 1F) caused more efficient apoptosis in Myc-ELAS1-expressing DU145 cells than in Myc-vector-expressing DU145 cells, as judged by the subG1 population (Figure 1G, 1H). These results suggest that ELAS1 also causes apoptosis in human DU145 cells.

### WT p53 is required to induce ELAS1-mediated apoptosis in SAS cells

**Figure 2:**
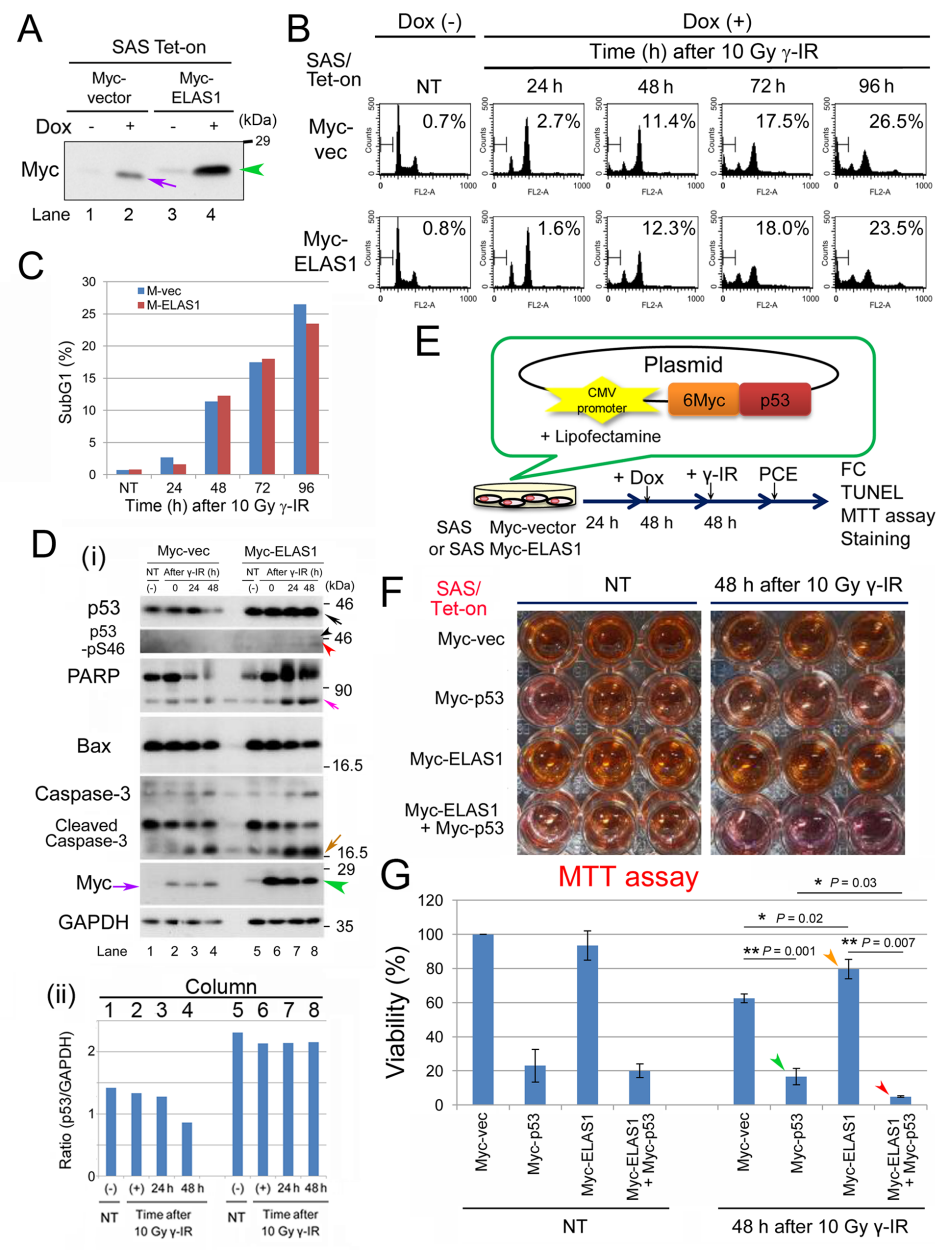
Exogenous expression of p53 rescues the apoptosis-inducing function of ELAS1 in p53-deficient SAS cells after dsDNA insults **(A)** Wb showing the successful establishment of SAS/Tet-On cells expressing Myc-vector or Myc-ELAS1 in the absence (-) or presence (+) of Dox. The purple arrow and green arrowhead indicate the Dox-inducible bands for Myc protein and Myc-ELAS1 protein, respectively. **(B)** FC analysis. SAS/Tet-On cells stably expressing Myc-vector alone or Myc-ELAS1 were treated with 10 Gy γ-IR for the indicated duration (h) in the presence of Dox. Percentages correspond to the sub-G1 population of cells. **(C)** The bar graphs show the percentages of subG1 cells as determined by FC shown in Figure. 2B. **(D)** (i) Wb examining expression of the indicated proteins. Black, pink, ocher, and purple arrows indicate bands for p53 (truncated size), PARP, cleaved caspase-3, and Myc-vector-derived Myc proteins, respectively. Black, red, and green arrowheads indicate bands for p53 (native size), p53 (truncated size), and Myc-ELAS1 proteins, respectively. (ii) Bar graph indicating the relative band intensity of p53 versus GAPD in Figure 5D-i. **(E)** A schematic presentation of the plasmid DNA construct that constitutively expressed 6Myc-tagged WT p53 (Myc-p53) proteins under the control of the CMV promoter when transfected into SAS/Tet-On Myc-ELAS1 cells using Lipofectamine. PCE, preparation of cell extract. This protocol was used for FC, TUNEL and MTT assays, and crystal violet staining. **(F)** SAS/Tet-on cells expressing Myc (Myc-vector), Myc-p53, Myc-ELAS1, or Myc-p53 and Myc-ELAS1 proteins were subjected to the cell viability test (MTT assay) at 48 h after 10 Gy γ-IR treatment. NT signifies non-treated cells used as a negative control. **(G)** The bar graphs show the percentage viability of SAS cells as determined by the MTT assay. Data represent the mean and SD of three independent experiments. Green and red arrowheads indicate the bars for SAS/Tet-on cells expressing Myc-p53 proteins in the absence and presence of Dox-dependent expression of Myc-ELAS1 at 48 h after 10 Gy γ-IR treatment.

**Figure 3:**
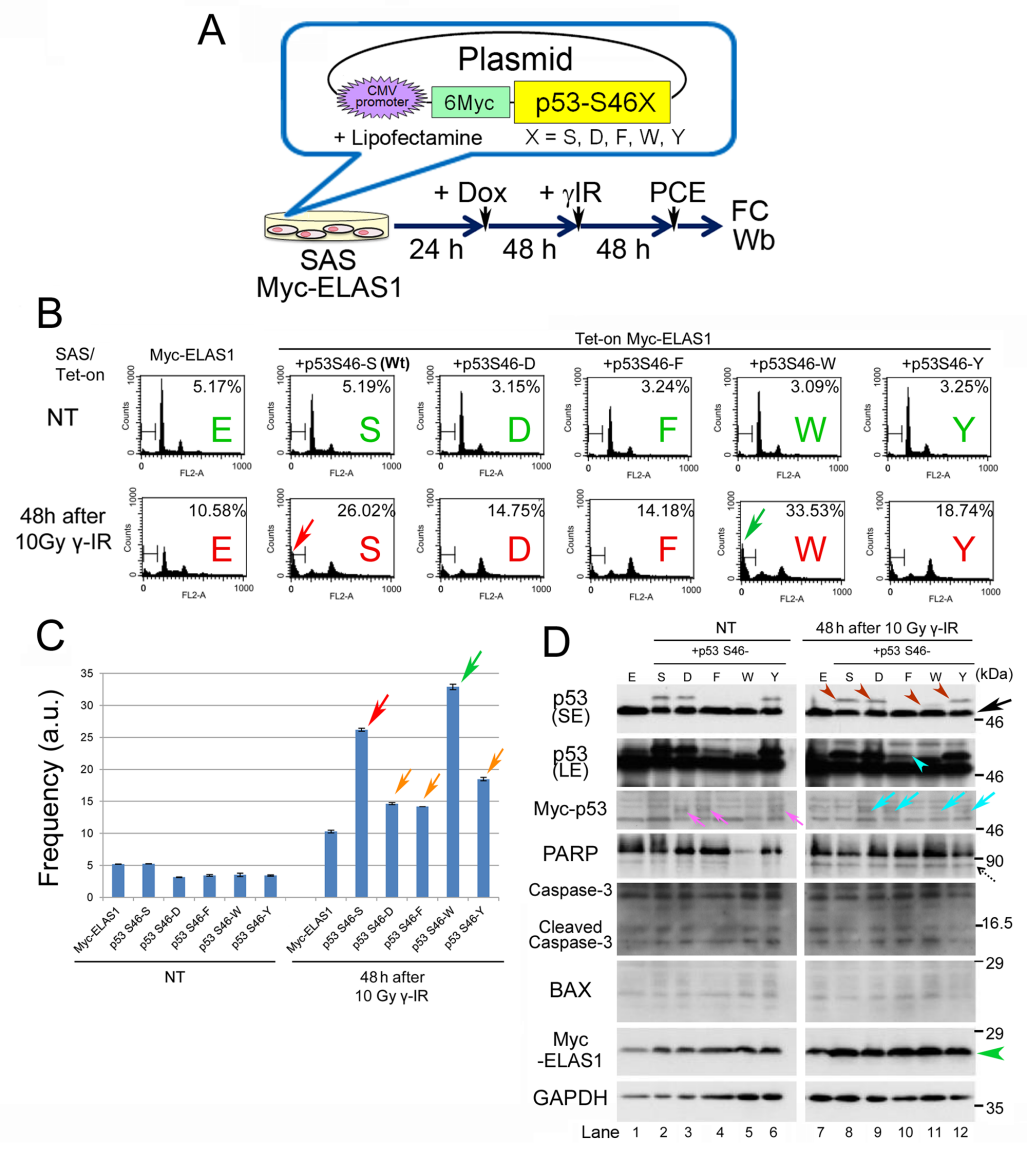
WT p53 is required to induce ELAS1-mediated apoptosis in SAS cells **(A)** A schematic presentation of plasmid DNAs that constitutively expressed Myc-p53-S46X (X = S, D, F, W, or Y) proteins under the control of the CMV promoter when transfected into SAS/Tet-On Myc-ELAS1 cells using Lipofectamine. PCE, preparation of cell extract. **(B)** FC analysis. SAS/Tet-On Myc-ELAS1 cells were transfected with Myc-p53-S46S (WT), -S46D, -S46F, -S46W, or -S46Y in the absence (NT) or presence of 10 Gy γ-IR treatment for 48 h. Red and green arrows indicate the increased subG1 population of Myc-p53-S46S- and -S46W-expressing SAS/Tet-On Myc-ELAS1 cells. **(C)** The bar graphs show the percentages of subG1 cells as determined by FC shown in Figure 3B **(D)** Wb to examine expression of the indicated proteins. Black, turquoise, and dotted arrows indicate bands for endogenous p53, exogenously expressed Myc-p53 (truncated size), and cleaved PARP, respectively. Brown, turquoise, and green arrowheads indicate bands for Myc-p53-S46X (X = S, D, W, or Y) proteins, Myc-p53-S46F observed only in the LE (long exposure) film, and Myc-ELAS1 protein, respectively. SE, short exposure.

We previously showed that ELAS1-mediated apoptosis in U2OS cells is due to increased stability of p53 and enhanced phosphorylation of p53-S46 [9]. Thus, we next determined whether ELAS1-mediated apoptosis occurs in p53-defective cancer cells such as SAS human tongue squamous-cell carcinoma cells. We also generated SAS/Tet-On cells that expressed Myc-vector or Myc-ELAS1 in a Dox-dependent manner. Their successful construction was confirmed by Wb (Figure 2A), as judged by the appearance of the Myc-ELAS1 band (green arrowhead), which migrated slower than the Myc-vector band (purple arrow). FC revealed that the frequency of the subG1 population at 24–96 h following 10 Gy γ-IR treatment differed little between cells expressing Myc-vector alone and those expressing Myc-ELAS1 in a Dox-dependent manner (Figures 3B, 3C). Interestingly, Wb identified a novel p53-pS46 band (black arrowhead in Figure 3D-i) at 24 and 48 h after 10 Gy γ-IR treatment in Myc-ELAS1-expressing SAS cells, although no such novel band was detected for p53 (top panel in Figure 3D-i). This band may have been derived from novel expression of the putatively silenced p53 gene in the other chromosome of the SAS genome. Specifically, Myc-ELAS1 may have induced the expression of the originally suppressed allelic counterpart of the p53 gene. The lower p53-pS46 band, the intensity of which peaked at 48 h (red arrowhead), migrated at the expected size of endogenous p53 (black arrow in the top panel), which was putatively derived from the p53_E336X mutant found in SAS cells. It remains elusive if the weak band intensity (red and black arrowheads) is due to a low level of p53-pS46 phosphorylation or the low sensitivity of the antibody. The increased intensity of this p53 band versus the GAPDH band in Myc-ELAS1-expressing SAS cells (columns 5–8 in Figure 3D-ii) compared with Myc-vec-expressing SAS cells (columns 1–4) suggests that ELAS1 also increases the stability of p53 in SAS cells, in addition to U2OS cells [9].

### Exogenously expressed p53 rescues ELAS1-meditated apoptosis in SAS cells

We next examined if exogenously expressed p53 proteins could rescue the attenuated apoptosis induction by ELAS1 in SAS/Tet-on cells. For this purpose, we transfected plasmid DNAs into Myc-vector- or Myc-ELAS1-expressing SAS/Tet-on cells; these plasmid DNAs were designed to express p53 proteins under the control of the cytomegalovirus (CMV) promoter (Figure 2E). Indeed, not only the MTT assay (Figure 2F, 2G) but also the TUNEL assay (Supplementary Figure 2), FC analysis (Supplementary Figure 3), and the cell growth speed assay using crystal violet staining (Supplementary Figure 4) showed that the level of apoptosis was higher when both Myc-p53 and Myc-ELAS1 proteins were expressed (red arrowheads) than when Myc-p53 alone (green arrowheads) or Myc-ELAS1 alone (orange arrowheads) was expressed at 48 h after treatment with 10 Gy γ-IR. These results suggest that ELAS1 efficiently promotes apoptotic death in SAS cells in the presence of exogenously expressed WT p53 protein. This apoptosis was dependent on the ELAS1 peptide but not on p53 because the level of apoptosis was significantly lower in Myc-p53-expressing cells than in Myc-ELAS1 plus Myc-p53-expressing cells. Although the values of the cell death indices differed, the induction of death in Myc-ELAS1 plus Myc-p53-expressing cells was reproducible.

Expression of Myc-ELAS1 alone appeared to have little effect on cell growth without DNA insults because there was little difference between the growth rates of SAS/Tet-On cells expressing Myc-vector and those expressing Myc-ELAS1, regardless of the presence of Dox (Supplementary Figure 5).

### Gain-of-function substitutions of p53-S46 efficiently rescue ELAS1-mediated apoptosis

Because the p53-S46F plasmid bearing a Ser-to-Phe substitution at codon 46 displays a gain-of-function phenotype that increases the transactivation activity of p53 protein [23], we aimed to induce more potent gain-of-function phenotypes by additionally preparing plasmid DNAs harboring substitutions at codon 46 such as p53-S46D, p53-S46F, p53-S46W, and p53-S46Y (Figure 3A). D mimics constitutive phosphorylation, while F, W, and Y have similar three-dimensional structures (Supplementary Figure 6). Indeed, when we compared their effects on apoptosis induction (Figure 3B), introduction of p53-S46S (WT) or p53-S46W increased the frequency of apoptotic cells by 2–3-fold compared with SAS cells expressing Myc-ELAS1 alone at 48 h after treatment with 10 Gy γ-IR (red and green arrows in Figure 3C). Unexpectedly, introduction of p53-S46D, p53-S46F, and p53-S46Y only slightly increased the subG1 population (orange arrows in Figure 3C). These results suggest that WT p53 successfully rescued the attenuated apoptosis induction by ELAS1 in SAS cells. Moreover, p53-S46W displayed a more potent gain-of-function phenotype than WT p53 (p53-S46S) and p53-S46F.

Wb confirmed the exogenous expression of p53-S46S, p53-S46D, and p53-S46Y proteins at similar levels using an anti-p53 antibody (top and second panels of Figure 3D) and an anti-Myc antibody (third panel). By contrast, the levels of the p53-S46W (which migrated faster than the other proteins) and p53-S46F proteins were lower than those of the other proteins in this order (Figure 3D). The reduced level of p53-S46F protein, which was probably due to its instability, may explain its unexpectedly modest apoptosis-inducing function (Figure 3C). By contrast, it is surprising that such a low level of p53-S46W displayed a remarkably enhanced apoptosis-inducing function (green arrow in Figure 3C). Notably, a faster migrating band was detected only in the case of p53-S46S, p53-S46D, p53-S46W, and p53-S46Y, either in the absence (lanes 2, 3, and 6 in Figure 3D) or presence (lanes 8, 9, 11, and 12 in Figure 3D) of γ-IR treatment, suggesting that apoptotic pathways were activated in these SAS cells. Wb also detected cleaved poly (ADP-ribose) polymerase (PARP) at 48 h after treatment with 10 Gy γ-IR (dotted arrow in Figure 3D). This band was generated in response to γ-IR treatment because it was also detected in cells without exogenous p53 expression (lane 7 in Figure 3D). Moreover, Wb failed to detect any increases or decreases in the levels of other apoptotic markers such as cleaved caspase-3 and BAX (Figure 3D). These results suggest that ELAS1 effectively induces cell death, as determined by the subG1 population, even in p53-defective cancer cells, provided that p53-S46S (WT) or p53-S46W (mutant) protein is exogenously expressed. Hereafter, we used p53-S46S (WT) protein alone for further study because p53-S46W is not a naturally occurring mutant.

### Simultaneous expression of Myc-ELAS1 and FLAG-p53 causes apoptosis in SAS cells

**Figure 4:**
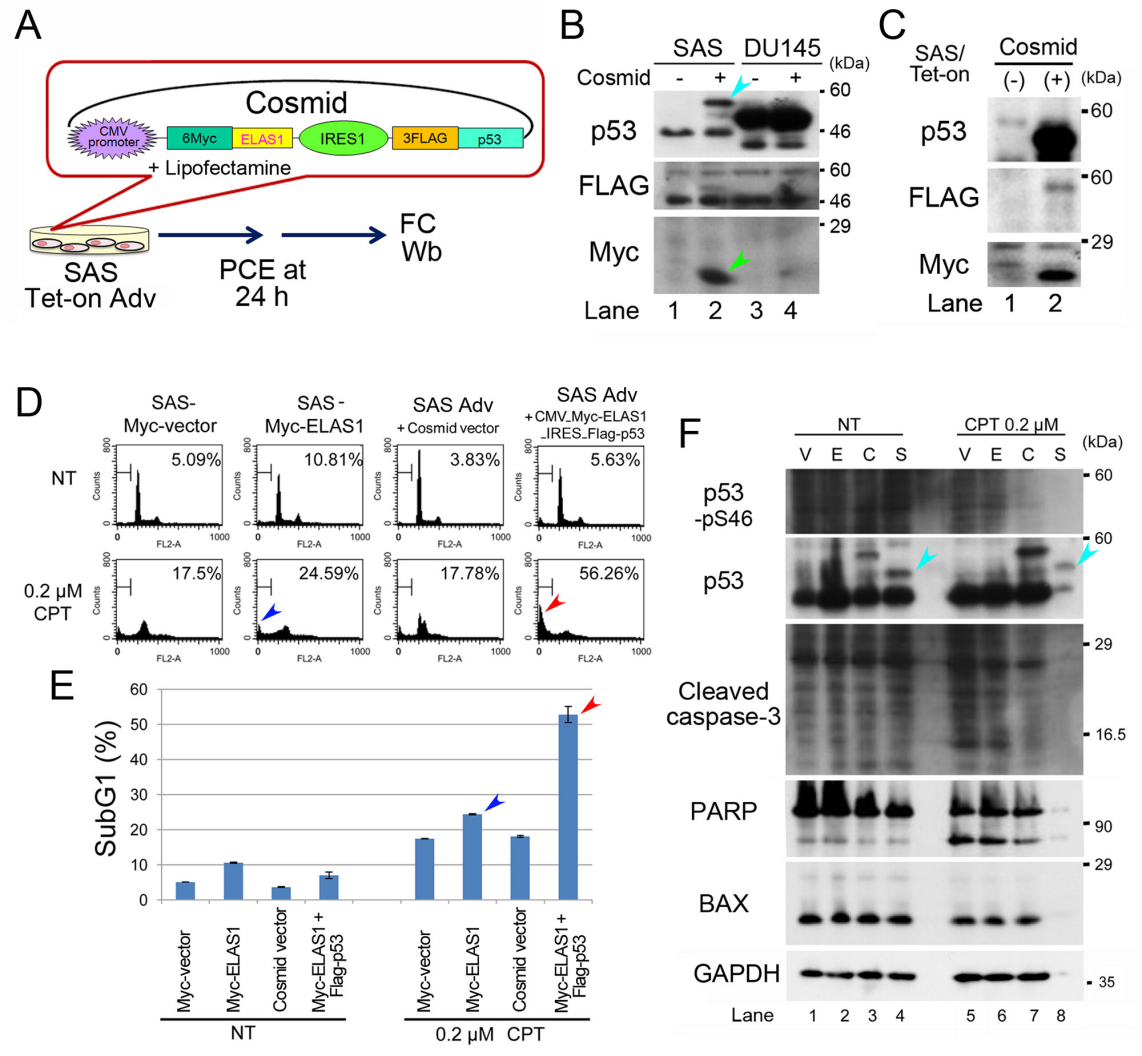
IRES-mediated co-translation of Myc-ELAS1 and FLAG-p53 rescues the apoptosis-inducing function of ELAS1 in p53-deficient SAS cells after CPT treatment **(A)** A schematic presentation of cosmid DNA that allows IRES-mediated co-translation of Myc-ELAS1 and FLAG-p53 in SAS/Tet-On Adv cells, and a protocol for the transfection experiment. PCE, preparation of cell extract. **(B)** Wb was performed to examine expression of the indicated proteins in the absence (-) or presence (+) of cosmid DNA. Turquoise and green arrowheads indicate bands for FLAG-p53 and Myc-ELAS1 derived from expression of cosmid DNA, respectively. **(C)** FC analysis. SAS/Tet-On Myc-vector, Myc-ELAS1, SAS Adv + cosmid vector, or SAS Adv + cosmid construct (CMV_6Myc-ELAS1_IRES_FLAG-p53) cells were transfected in the absence (NT) or presence of treatment with 0.2 μM CPT for 48 h. **(D)** The bar graphs show the percentages of subG1 cells as determined by FC. Red and blue arrows correspond to the subG1 peak of FC data shown in Figure 4C. **(E)** Wb to examine expression of the indicated proteins. Turquoise arrowheads indicate the band for FLAG-p53.

Next, we examined co-expression of Myc-ELAS1 and FLAG-p53 proteins through an internal ribosome entry site (IRES), which allows translation initiation in an end-independent manner by inserting their cDNAs into a cosmid vector (Figure 4A, Supplementary Figure 7). Indeed, IRES enabled the cosmid to express both Myc-ELAS1 and FLAG-p53 proteins at similar levels in SAS cells (turquoise and green arrowheads in Figure 4B). FC showed that the subG1 population after treatment with 0.2 μM CPT for 48 h was only slightly higher for SAS cells expressing Myc-ELAS1 than for SAS cells expressing Myc-vector alone (blue arrowheads in Figure 4C and Figure 4D). However, the subG1 population of SAS advanced (Adv) cells expressing Dox-inducible FLAG-p53 and Myc-ELAS1 following transfection of cosmid DNA was 3-fold larger than that of cells expressing the cosmid vector alone (red arrowheads in Figure 4C and Figure 4D). Wb confirmed the exogenous expression of Myc-ELAS1 and FLAG-p53 in NT conditions (Figure 4E), in which the expression levels of cleaved caspase-3, PARP, and BAX were almost the same in cells expressing Myc-vector (V), Myc-ELAS1 (E), and the cosmid vector (C). However, Wb was not useful to examine the levels of apoptotic marker proteins in Myc-ELAS1/FLAG-p53-expressing SAS cells treated with 0.2 μM CPT (lane 8 in Figure 4E) because most proteins were degraded due to cell death (56.26%). Nonetheless, these results suggest that IRES-mediated simultaneous expression of Myc-ELAS1 and FLAG-p53 is beneficial to rescue the apoptosis-inducing function of ELAS1 even in p53-deficient cells.

### ELAS1 does not cause apoptosis in normal KD cells

**Figure 5:**
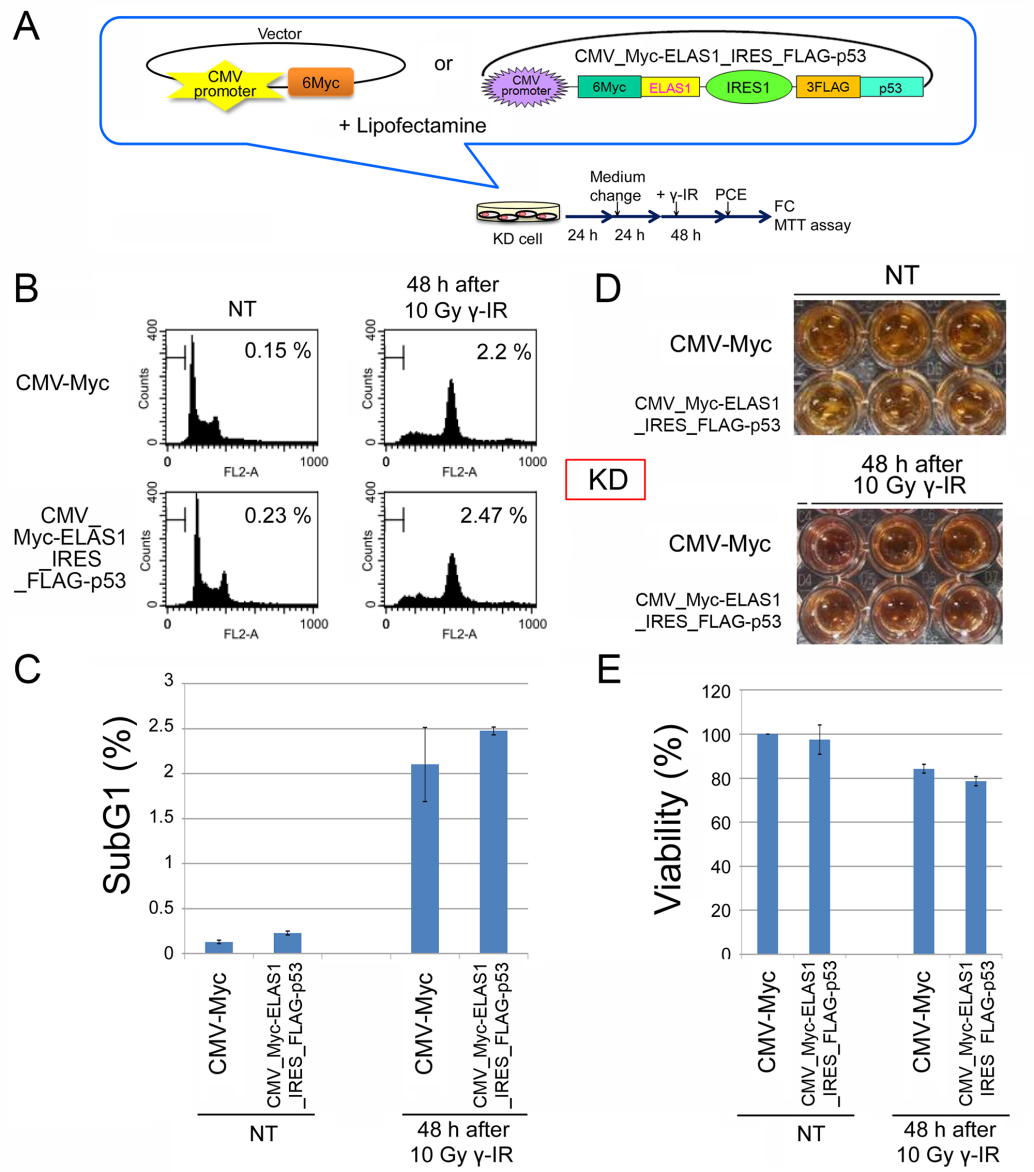
ELAS1-mediated apoptosis does not occur in normal human fibroblast KD cells **(A)** A schematic presentation of the plasmid or cosmid DNA that constitutively expressed 6Myc-tagged WT p53 (Myc-p53) proteins or IRES-mediated Myc-ELAS1 and FLAG-p53 proteins under the control of the CMV promoter when transfected into normal human fibroblast KD cells using Lipofectamine. PCE, preparation of cell extract. This protocol was used for FC and the MTT assay. **(B)** Typical FC patterns are shown with percentages of sub-G1 cells. Cells were stained with propidium iodide and the cell cycle profiles were determined by FC. Data were obtained at 48 h after 10 Gy γ-IR treatment. NT means non-treated cells used as a negative control. **(C)** The bar graph shows the percentage of sub-G1 cells. Data represent the mean and SD of three independent experiments (20,000 cells per experiment). **(D)** The cell viability assay revealed little ELAS1-mediated apoptosis in KD cells after γ-IR treatment. KD cells expressing Myc (Myc-vector) or IRES-mediated Myc-p53 and Myc-ELAS1 proteins were subjected to a cell viability test (MTT assay) at 48 h after 10 Gy γ-IR treatment. NT signifies non-treated cells used as a negative control. **(E)** The bar graphs show the percentage viability of KD cells as determined by the MTT assay. Data represent the mean and SD of three independent experiments.

To test if ELAS1 also induces apoptosis in normal cells, we performed similar experiments using KD cells, a normal human fibroblast cell line. We used the IRES system to simultaneously express the Myc-ELAS1 and FLAG-p53 proteins (Figure 5A). Unlike in SAS cancer cells, there was little difference in the subG1 population determined by FC (Figure 5B, 5C) and the cell viability assay (Figure 5D, 5E) between cells expressing the vector alone and those expressing both Myc-ELAS1 and FLAG-p53. We performed the analyses at 48 h because plasmid gene expression is optimal at this time point using Dox and Lipofectamine systems. Additional time points are useful for observing the influence of γ-IR, if gene expression is maintained. Thus, ELAS1-mediated apoptosis occurs only in cancer cells, not in normal KD cells.

### Use of an adenovirus to introduce ELAS1 and p53 into cancer cells

**Figure 6:**
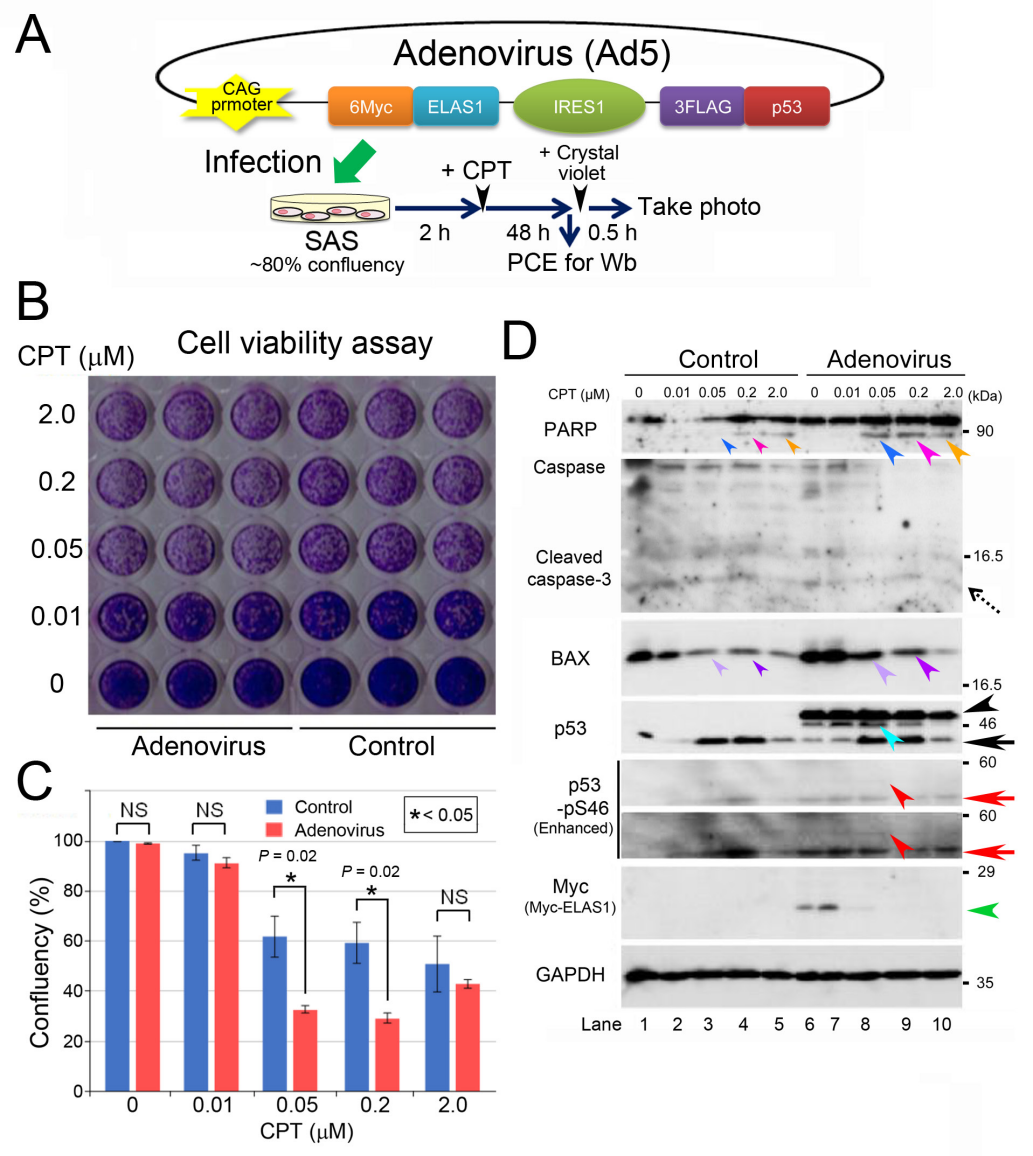
An adenovirus harboring Myc-ELAS1_IRES_FLAG-p53 causes apoptotic death of SAS cells after CPT treatment **(A)** A schematic presentation of adenoviral DNA that allows IRES-mediated co-translation of Myc-ELAS1 and FLAG-p53 in SAS cells under the control of the CAG promoter, and a protocol for the transfection experiment. PCE, preparation of cell extract. **(B)** A photograph of the culture plate after a cell viability assay using crystal violet. Live cells, but not dead cells, were stained light purple by crystal violet. Control means SAS cells without adenovirus infection. **(C)** Confluency analysis of the data shown in Figure 4B. Statistical significance was calculated using three independent assays. **(D)** Wb to show that an adenovirus harboring Myc-ELAS1_IRES_FLAG-p53 causes apoptotic cell death. Dotted, black, and red arrows indicate cleaved caspase-3, endogenous p53, and endogenous p53-pS46, respectively. Turquoise, red, and green arrowheads indicate bands for IRES-mediated FLAG-p53, FLAG-p53-pS46, and Myc-ELAS1, respectively. NS, not significant.

For the clinical application of ELAS1, we explored the usefulness of an adenovirus as a tool to efficiently introduce the ELAS1 peptide into cancer cells. We inserted the above described cosmid vector harboring Myc-ELAS1+IRES+FLAG-p53 cDNA into the adenovirus 5 (Ad5) genome equipped with the CAG promoter (CAG means **C**MV early enhancer fused to chicken beta-**a**ctin promoter and rabbit beta-**g**lobin poly A site) (Figure 6A). We infected SAS cells with this adenovirus and performed the cell viability assay with or without CPT treatment (Figure 6B) according to the time schedule depicted in the bottom panel of Figure 6A. Quantification of confluency using GIMP and Excel software revealed that adenovirus-infected SAS cells conspicuously died in the presence of 0.05 and 0.2 μM CPT compared with the negative control (without adenovirus infection), suggesting the successful expression of apoptosis-inducing ELAS1 in adenovirus-infected SAS cells (Figure 6C).

To confirm the IRES-mediated expression of Myc-ELAS1 and FLAG-p53 proteins in adenovirus-infected SAS cells, we prepared extracts of these cells for Wb. The level of PARP cleavage was higher after adenovirus infection in the presence of 0.05 and 0.2 μM CPT than in the negative control (compare the band intensities between the small and large blue/pink arrowheads in the top panel of Figure 6D). The band intensities in samples treated with 2 μM CPT were similar with and without adenovirus infection, which is probably due to apoptosis induced by CPT (orange arrowheads). Moreover, BAX expression was increased by adenovirus infection, as judged by the difference in band intensities between the small and large violet/purple arrowheads in the BAX panel of Figure 6D. By contrast, the level of cleaved caspase-3 (dotted arrow) was almost unaltered. The expression levels of FLAG-p53 (turquoise arrowhead) and Myc-ELAS1 (green arrowhead) peaked following treatment with 0.01 and 0.05 μM CPT, respectively. Their band intensities decreased upon treatment with higher concentrations of CPT, probably due to protein degradation during cell death. Levels of FLAG-p53-pS46 (red arrowheads) and endogenous p53-pS46 (red arrows) also peaked following treatment with 0.05 μM CPT. These results suggest that adenovirus-mediated expression of Myc-ELAS1 and FLAG-p53 proteins caused apoptosis in p53-defective SAS cells.

### Expression of ELAS1 is effective for treatment of orthotopic tongue tumors in mice

To examine if implantation of ELAS1-expressing cancer cells prolongs the survival of mice due to the apoptosis-inducing function of ELAS1, we inoculated SAS/Tet-On cells, which expressed Dox-inducible Myc-vector or Myc-ELAS1, into the left side of the tongue of each nude mouse and monitored their weight every 2 or 3 days (Supplementary Figure 8A). Nude mice harboring Myc-ELAS1-expressing SAS cells lived longer than nude mice harboring Myc-vector-expressing SAS cells, as evaluated by the log-rank test using Kaplan–Meier survival curves (Supplementary Figure 8B). Mouse body weight provides a better indicator of the effect of ELAS1 on orthotopic tongue tumors, because tumor growth in the tongue can potentially cause eating disorders and reduce body weight. Indeed, the body weight of mice implanted with Myc-ELAS1-expressing cells was higher than that of mice implanted with Myc-vector-expressing cells at 12–28 days after implantation (Supplementary Figure 8C), suggesting that the former mice remained healthier than the latter mice. Immunohistochemistry demonstrated efficient expression of Myc and Myc-ELAS1 proteins in tumors formed by Myc-vector- and Myc-ELAS1-expressing SAS/Tet-On cells, respectively. These results suggest that mice implanted with Myc-ELAS1-expressing SAS cells tended to have a higher survival rate following treatment with irinotecan, a CPT analogue that is less toxic but maintains its anti-cancer effects. We performed a similar experiment by monitoring the mice over a longer period until even mice implanted with Myc-ELAS1-expressing SAS cells started to die. This confirmed the reproducibility of the result (Supplementary Figure 9A, 9B).

Interestingly, when we performed a similar experiment in the absence of irinotecan injection, mice implanted with Myc-ELAS1-expressing SAS cells also tended to have a higher survival rate than mice implanted with Myc-vector-expressing SAS cells (Supplementary Figure 9C). This suggests that the DSB stimulus is not required for ELAS1 function *in vivo*, which may open up the possibility of ELAS1 usage at the bedside in the future.

### Minimum fragment of ELAS1 that retains its apoptosis-inducing function

**Figure 7:**
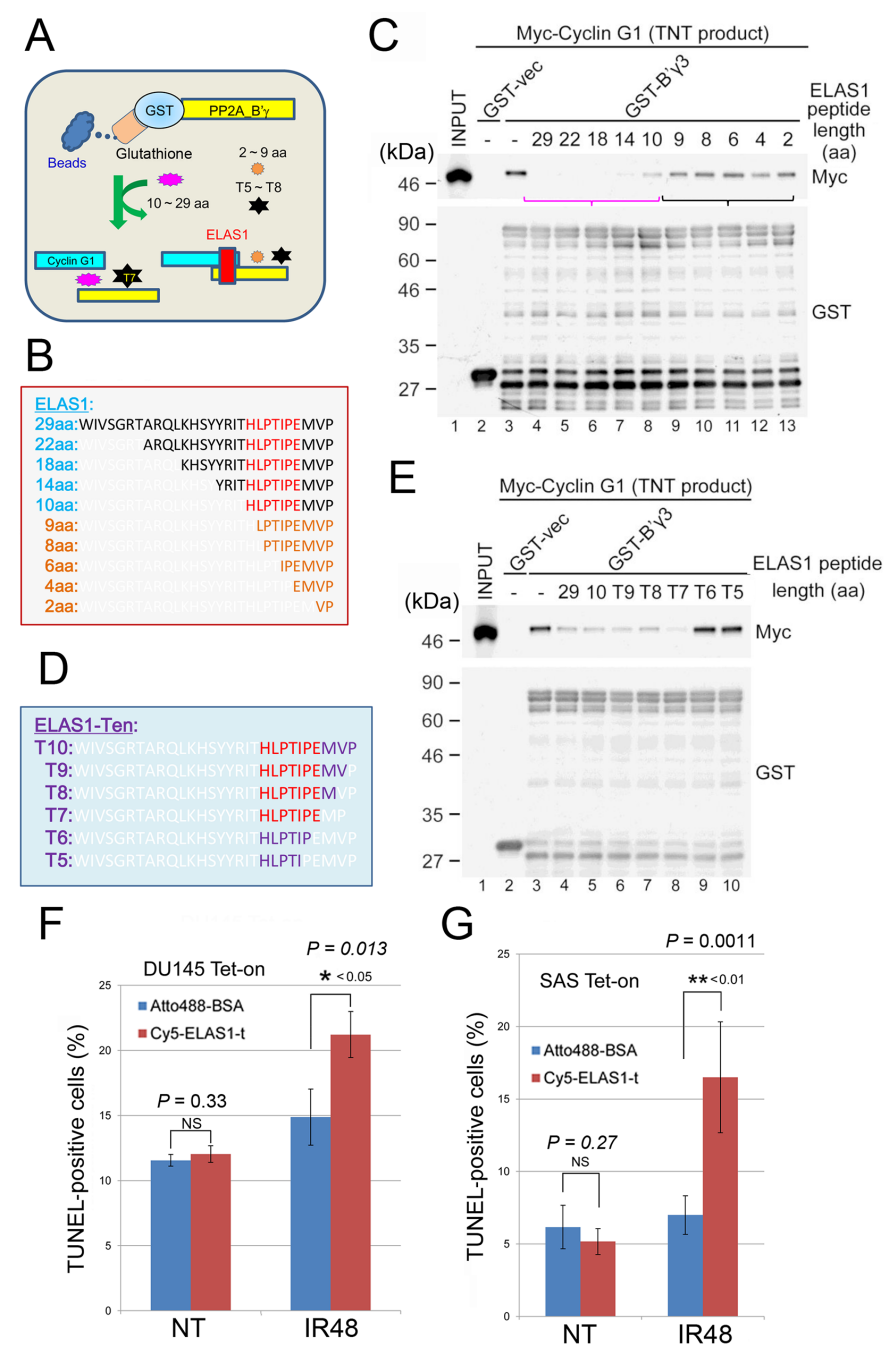
Determination of the minimum fragment of ELAS1 that retains its original apoptosis-inducing function **(A)** A schematic presentation of the experimental protocol. **(B–E)** Amino acid sequences of ELAS1-derived peptides truncated from its N-terminus (B) or ELAS1-t-derived peptides truncated from its C-terminus (D) that were used for the inhibition experiments. Wb to examine the inhibitory effect of ELAS1-derived peptides on the CycG1-B’γ association using an anti-Myc antibody. (C, E) Wb with an anti-GST antibody to show the use of equal amounts of GST-B’γ3 protein. **(F, G)** The bar graph shows the percentage of TUNEL-positive cells in the microscopy images (Supplementary Figure 10). The data represent the means and SD of three independent experiments (200 cells were counted per experiment).

The original ELAS1 peptide (29 aa) might be too long for direct use as a peptide drug. To examine if a shorter ELAS1 peptide retains its original apoptosis-inducing function, we chemically synthesized peptides shorter than 29 aa. We first examined their inhibitory effect on the CycG1-B’γ association by including them in a reaction mixture containing GST-B’γ3, the largest alternative splicing product of the B’γ gene, and Myc-CycG1 (Figure 7A). ELAS1 peptides containing 22, 18, or 14 aa (Figure 7B) efficiently inhibited the CycG1-B’γ association as effectively as the original 29 aa ELAS1 peptide (lanes 4–7 in Figure 7C). By contrast, ELAS1 peptides containing 9, 8, 6, or 2 aa (Figure 7B) had little inhibitory effect on the CycG1-B’γ association (lanes 9–13 in Figure 7C). Because the ELAS1 peptide containing 10 aa (ELAS1-Ten or ELAS1-t) had a modest inhibitory effect (lane 8 in Figure 7C), we further synthesized peptides by shortening ELAS1-t from its C-terminus (Figure 7D). T10, T9, T8, and T7, but not T6 or T5, had an inhibitory effect on the CycG1-B’γ association (lanes 5–8 in Figure 7E). Thus, we selected ELAS1-t for further analysis.

We next examined if Cyc5-tagged ELAS1-t could induce apoptosis in DU145 and SAS cells because we previously demonstrated that Cyc5-ELAS1 induces apoptosis in U2OS cells [9]. Indeed, the TUNEL assay suggested that Cyc5-ELAS1-t efficiently caused apoptosis in both DU145 (Figure 7F) and SAS (Figure 7G, Supplementary Figure 10) cells. Notably, exogenously expressed p53 was not required to induce apoptosis in SAS cells, suggesting that ELAS1-t is a useful peptide drug regardless of the presence of native p53. Taken together, ELAS1 peptides longer than 10 aa would be useful as peptide drugs.

## DISCUSSION

Our previous study [9] raised three concerns. The first is the small number of osteosarcoma patients. Here, we eliminated this concern by showing efficient ELAS1-mediated apoptosis induction in both prostate cancer DU145 cells (Figure 1, Supplementary Figure 1, and Figure 7F) and tongue cancer SAS cells (Figures 2, 3, 4, 6, and 7 and Supplementary Figures 2 –4). The second concern is that ELAS1 is dependent on increased stability of p53 and enhanced phosphorylation of p53-S46 [9] because ELAS1 may not cause apoptosis in p53-deficient cancer cells. Here, we diminished this concern by showing that ELAS1 promoted apoptotic cell death in the presence of exogenously expressed WT p53 proteins (Supplementary Figures 2– 4). The third concern is that ELAS1 is toxic to normal cells. This concern was removed by showing that ELAS1 caused little apoptosis in normal KD cells (Figure 5). The healthy growth of CycG1- and CycG2-double-knockout mice [13] also supports the safety of ELAS1, which inhibits a function of CycG1 [9].

Moreover, we identified p53-S46W as a novel gain-of-function mutant (Figures 3B–D), which may be useful for clinical application of ELAS1. In TUNEL (Supplementary Figure 2) and FC (Supplementary Figure 3) analyses, p53 appeared to be the major cause of apoptosis. By contrast, ELAS1 expression was the major cause of apoptosis in the MTT assay (Figure 2F–G) and crystal violet staining analysis (Supplementary Figure 4). In general, the population of dead cells is measured by the TUNEL and FC assays, in which the effect of p53 expression was more apparent than that of ELAS1. By contrast, the MTT assay and crystal violet staining quantify the frequency of surviving cells (x), whose reciprocal number (1/x) indicates non-surviving (almost dead) cells. This means that simultaneous expression of ELAS1 and p53 reduced the frequency of surviving cancer cells more efficiently than expression of p53 alone. Furthermore, mice implanted with Myc-ELAS1-expressing SAS cells tended to have a higher survival rate than mice implanted with Myc-vector-expressing SAS cells (Supplementary Figure 8 and Supplementary Figure 9).

Our results also showed that IRES-mediated simultaneous expression of Myc-ELAS1 and FLAG-p53 caused efficient apoptosis in SAS cells (Figure 4). This result prompted us to prepare a recombinant adenovirus to introduce ELAS1 and p53 into cancer cells. As expected, we observed IRES-mediated expression of Myc-ELAS1 and FLAG-p53 proteins in adenovirus-infected SAS cells in the presence of CPT (Figure 6). Based on these results, we propose that oncolytic viruses such as recombinant adenovirus 5 and herpes simplex virus type 1 (HSV-1) may be ideal vehicles to introduce the ELAS1 peptide into cancer cells and may activate its apoptosis-inducing function and thereby enhance their oncolytic potential [24]. Telomelysin (OBP-301) is a recombinant adenovirus that is not expressed in normal cells but is highly expressed in cancer cells [25]. An IRES-mediated p53-expressing oncolytic adenovirus (OBP-702) induces profound apoptosis in OBP-301-resistant osteosarcoma cells [26]. Therefore, it would be preferable to replace the p53 construct with ELAS1_IRES_p53 (Figure 5 and Figure 6) to provide Telomelysin with greatly augmented oncolytic activity. HSV1_G47Δ, which replicates only in cancer cells, would also be a good vehicle to carry ELAS1 specifically into cancer cells [27].

Finally, we investigated the minimum fragment of the ELAS1 peptide that retains its original apoptosis-inducing function. Because the above described experiments were based on cancer cells pre-transduced with the ELAS1 gene, the apoptotic effect was observed when the transduction efficiency was nearly 100%. By contrast, in the real therapeutic context, the *in vivo* transduction efficiency does not reach that high even with using an oncolytic virus, which may make it difficult to expect meaningful therapeutic effects of ELAS1 in real therapeutic settings. To circumvent this problem, we directly incorporated ELAS1 peptide into U2OS cells in our previous report [9] because the amounts of peptides used at the bedside could be greatly increased if an efficient drug delivery system (DDS) is developed, which may rescue the low transduction efficiency. Although peptide-based therapies are associated with disadvantages in general such as poor membrane permeability and metabolic instability, there are examples of successful small peptides such as bortezomib (2 aa), octreotide (8 aa), leuprorelin and goserelin (9 aa), and glatiramer acetate (10 aa) [28]. For the efficient design of ELAS1 in clinical applications, it is essential to consider not only the stability of the peptide but also the cost of its synthesis. For this purpose, the 29 aa ELAS1 was shortened. Our results are encouraging because a chemically synthesized ELAS1 peptide that was shortened to 10 aa from its N-terminus (ELAS1-t) also had an inhibitory effect on the CycG1-B’γ association *in vitro* (Figure 7C). ELAS1-t effectively caused apoptotic death of DU145 (Figure 7F) and SAS (Figure 7G) cells. These results will encourage us to develop an appropriate DDS for the ELAS1-t peptide in future experiments to increase its usefulness as a peptide drug. They also suggest that the functional core domain of ELAS1 (29 aa) as an inhibitor of the CycG1-B’γ association resides in its C-terminus, and is present in ELAS1-t. Because the other missing 19 aa are not required for its function, ELAS1-t is expected to have increased selectivity. Taken together, our results suggest that ELAS1 might be therapeutically useful if it is delivered *via* an adenovirus or used directly as a peptide drug.

## MATERIALS AND METHODS

Further information can be found in Supplementary Materials.

### Antibodies

Antibodies raised against various proteins were purchased from the indicated commercial sources. The monoclonal antibodies were as follows: anti-Myc and anti-GST (MBL), anti-FLAG (Sigma-Aldrich), anti-p53 (Santa Cruz Biotechnology), and anti-GAPDH (Fitzgerald Industries International). The polyclonal antibodies were as follows: anti-FLAG (Sigma-Aldrich); anti-caspase-3, anti-PARP, and anti-p53-pS46 (Cell Signaling Technology); and anti-BAX, anti-p53, and anti-Myc (MBL).

### Peptides

Peptides with N-terminal acetylation (Figure 6B, 6D) and the Cy5C_ELAS1-t peptide (Figure 7F, 7G and Supplementary Figure 10) were synthesized and purified (>90%) by GenScript.

### Statistics

Statistically significant differences were determined by the Student’s t-test using Microsoft Excel 2013 software (Microsoft). The data are expressed as means ± SE. P values <0.01 (**) and <0.05 (*) were considered to be statistically significant. Statistical analyses of survival curves (Supplementary Figures 8 and 9) were performed using the nonparametric Kaplan–Meier method. Differences between the survival curves were examined using pairwise log-rank tests (Mantel-Cox). Relationships between the mean survival times of mice bearing SAS/Tet-on cells expressing Myc-vector or Myc-ELAS1 at different time points (days) were analyzed using Microsoft Excel.

## SUPPLEMENTARY MATERIALS FIGURES


