# Supplementary material for: ELAS1 induces apoptotic death in adenocarcinoma DU145 and squamous-cell carcinoma SAS cancer cells, but not in normal KD cells
Source: Oncotarget. 2017 Aug 24;8(49):85868–82. doi: 10.18632/oncotarget.20696 (PMC5689653; doi:10.18632/oncotarget.20696)
Supplement: Supplementary file 1 [file oncotarget-08-85868-s001.pdf]

ELAS1 induces apoptotic death in adenocarcinoma DU145 and squamous-cell carcinoma SAS cancer cells, but not in normal KD cells

SUPPLEMENTARY MATERIALS

Plasmid DNA constructs

CycG1 cDNAs were inserted into the *Asc*I and *Not*I sites of the following expression vectors: pCMV-6Myc for 6Myc-tagged proteins, p3FLAG for 3Flag-tagged proteins, and pGST6P for GST-fusion proteins, as described previously [35, 36]. The construction of Myc-ELAS1 and GST-PP2A B'γ3 was described previously [9]. For the Tet-ON inducible expression system, pTet-On Advanced and pTRET3-6Myc vectors were used (Clontech). pTRET3-6Myc-ELAS1 was constructed by ligating the ELAS1 fragment into the *Asc*I and *Not*I sites of the pTRET3-6Myc vector[9]. All amplified and synthesized sequences were confirmed by DNA sequencing. To construct plasmid DNAs that expressed p53-S46S, p53-S46D, p53-S46F, p53-S46W, and p53-S46Y proteins, double-stranded DNAs (dsDNAs) harboring point mutations (see Figure 2E and Figure S1) were chemically synthesized by GenScript and inserted into pTRET3-6Myc-p53 plasmid DNA via *Acl*I and *Psh*A1.

Cell culture and transfection

DU145 human prostate cancer cells, SAS human tongue cancer cells, and 293T human embryonic kidney cells were maintained in Dulbecco's modified Eagle's medium (DMEM; Sigma-Aldrich, D5796) supplemented with 10% fetal bovine serum (FBS; HyClone, SH30910.03) and 100 U/ml penicillin/100 µg/ml streptomycin (Nacalai Tesque, #26253-84).

Preparation of DU145 and SAS Adv cells and their Tet-ON inducible cell lines

To prepare tetracycline-inducible DU145 (DU145/Tet-On cells) or SAS (SAS/Tet-On cells) cells, DU145 or SAS cells were transfected with the pTet-On Advanced Vector plasmid (Clontech) and incubated in DMEM supplemented with 10% FBS or Tet system-approved FBS (Clontech, 631101), penicillin/streptomycin, and 0.8 mg/ml G418. Thereafter, several single colonies were selected for further examination.

The pTRET3-6Myc vector was constructed by inserting a multi-cloning site linker containing 6Myc-tag (*Bam*HI-*Hin*dIII-*Cla*I-6Myc-*Asc*I-*Eco*RV-*Not*I-*Sal*I) into the *Bam*HI and *Sal*I sites of the pTRE-Tight vector (Clontech). DU145/Tet-On and SAS/Tet-On cells were co-transfected with each of the pTRET3-6Myc plasmids (including the empty Myc-vector and Myc-ELAS1) together with Linear Hygromycin Marker (Clontech) using Lipofectamine and PLUS reagents according to the manufacturer's instructions (Invitrogen). Transfected cells were serially diluted and selected with culture medium containing hygromycin (0.2 mg/ml). Single colonies were isolated and positive clones were confirmed by examining the expression patterns in the presence or absence of Dox (1 µg/ml).

γ-IR and drug treatment

Cells were cultured on coverslips and treated with 10 Gy γ-IR using a Gammacell 40 Exactor (Best Theratronics) or the following drugs: 20 µM irinotecan, 100 µg/ml 5-FU, or 10 µM CPT. After treatment with these drugs for 48 h, the culture medium was removed, the cell plate was washed with calcium- and magnesium-free PBS [PBS (-)], and cells were subjected to FC and Wb analyses. Irinotecan and CPT were purchased from Sigma-Aldrich.

Peptide transfection

The Cy5-labeled ELAS1-t peptide was introduced into DU145_Adv or SAS_Adv cells plated on glass coverslips with a JBS Protein Transduction Kit (Jena Bioscience) and incubated for 24 h (NT sample). Atto488-BSA was used as a positive control for successful transduction. At 3 h after peptide transfection, IR48 samples were treated with 10 Gy IR and then incubated for 48 h. The cells on coverslips were washed two times with PBS (-), three times with glycine buffer, and two times with PBS (-), and then fixed by sequential incubation with 4% formaldehyde prepared in PBS (-), 0.1% Triton X-100 prepared in PBS (-), and 0.05% Tween-20 prepared in PBS (-). After washing, apoptotic cells were detected by the TUNEL assay and observed using a laser scanning confocal microscope (FV10i; Olympus).

Wb analysis

Total cell lysates and immunoprecipitates were resolved by SDS-PAGE and then transferred to PVDF membranes. The membranes were blocked with TBS-T (20 mM Tris-HCl [pH 7.5], 150 mM NaCl, and 0.05% Tween-20) containing 5% skimmed milk, and then probed with the relevant antibodies. Immunoreactive protein

bands were visualized using Western Lightning Plus-ECL (PerkinElmer, NEL105001EA) or Western Lightning ECL Pro (PerkinElmer, NEL122001EA).

GST pull-down and inhibition assay

To prepare GST-purified protein baits, GST-fused CycG1 and B'γ proteins expressed in the *E. coli* PR745 strain were lysed by sonication and purified using Glutathione Sepharose 4B (GE Healthcare). An *in vitro* transcription/translation system (TnT T7 Quick Coupled System, Promega) was used to prepare lysate prey, in which pCMV6myc-CycG1 or PP2A_B'γ was added to rabbit reticulocyte lysate (TnT Quick Master Mix) containing 20 µM methionine and incubated at 30°C for 90 min. Protein–protein interactions were examined by adhering the GST-fusion proteins to Glutathione Sepharose 4B, followed by incubation with the TnT lysates expressing 6Myc-tagged proteins by rotating at 4°C overnight. The bound proteins were eluted by boiling in Laemmli buffer after washing with 1 ml of N04 buffer containing 5-fold-concentrated protease inhibitors and then analyzed by SDS-PAGE and western blotting. For the peptide inhibition assay, the GST pull-down assay was conducted in the presence or absence of the indicated peptides (Figure 6), followed by Wb using an anti-Myc or anti-GST (loading control) antibody.

FC analysis

Incubated cells were washed with PBS (-), collected by trypsinization and centrifugation, resuspended, washed twice in PBS (-), and fixed with 70% ethanol. Then, cells were treated with propidium iodide (20 µg/ml) and RNase A (200 µg/ml). Cell sizes and DNA contents were measured using a FACSCalibur instrument (Becton Dickinson) and analyzed using CellQuest software (BD Bioscience).

Cell viability assay

To examine the frequency of cell viability, we performed the methyl tetrazolium (MTT) assays. SAS or KD cells were plated at 96-well plates (1.0×10^4 cells/well) and incubated overnight. Then, 40 ng/well of DNA were transfected into SAS or KD using lipofectamine (Thermo Fisher Scientific). After 24 h of transaction, Dox was added and incubated for further 48 h. Then, cells were exposed to 10 Gy γ-IR. After additional 48 h of incubation, MTT assays were performed according to the manufacturer's protocol (Dojinbo). Briefly, 10 µl of cell counting Kit-8 (Dojindo, http://www.dojindo.com/store/p/456-Cell-Counting-Kit-8.html) was added into cells, which were incubated for an additional 3 h. Then, absorbance at 450 nm was measured with a plate reader. The cell viability was determined by (absorbance of cells)/(absorbance of non-treated vector cells) × 100. Non-treated vector cells were used as a standard.

TUNEL assays

DU145 and SAS cells plated and incubated on cover glasses were fixed by sequential incubation with 4% formaldehyde prepared in PBS (-) for 25 min at 4°C, 0.2% Triton X-100 prepared in PBS (-) for 5 min, and 0.05% NaN_3 prepared in PBS (-) for 10 min at room temperature. The DeadEnd Colorimetric TUNEL System (Promega), which end-labels fragmented DNA, was used to detect apoptotic cells. Then, the fixation solution was washed out and cells were incubated with recombinant terminal deoxynucleotidyl transferase in the presence of biotinylated nucleotides for 1 h at 37°C. Next, cells were washed with PBS (-), treated with 2× SSC for 15 min at room temperature in the dark, stained with Hoechst 33258 (Sigma), and observed using a BX51 microscope (Olympus).

Preparation of cosmid DNA and recombinant adenovirus constructs

dsDNAs encoding Kozak+6Myc or *Asc*I+6Myc-ELAS1+IRES+3Flag-p53_*Not*I were chemically synthesized by TaKaRa Inc. (http://www.takara-bio.co.jp/) or GenScript Inc. (http://www.genscript.com/), respectively (Figure S3). They were inserted into the *Smi*I site of pAxCAwtit2 (a cosmid vector for recombinant adenovirus construction) using the In Fusion technique (TaKaRa code Z9648N) with the aid of PCR amplification. Successful cloning of the correct construct (Kozak+6Myc-ELAS1+IRES+3Flag-p53) was confirmed by restriction enzyme digestion (5'/*Cla*I/*Smi*I/*Psi*I/*Spe*I/*Xba*I/*Cla*I/3') and DNA sequencing. These experiments were performed by TaKaRa Inc.

Preparation of adenoviruses and infection of SAS cells

A recombinant adenovirus expressing the Kozak+6Myc-ELAS1+IRES+3Flag-p53 construct under the control of the CAG promoter was prepared by TaKaRa Inc., sent to Unitech Inc. (http://www.uniqtech.co.jp/), and amplified in 293 cells (ATCC, CRL-1573). SAS cells were infected with the amplified adenovirus at a multiplicity of infection of 1.0. A cell viability assay was conducted using crystal violet according to the protocol shown in the bottom panel of Figure 4A. These experiments were performed by Unitech Inc.

Confluency analysis

Extraction and measurement of the areas of adherent culture cells in the cell viability assay (Figure 4B) were performed using GIMP software, ver. 2.8.14 (GIMP Development Team).Quantification of confluency and statistical significance was performed using Microsoft Excel 2013 software (Microsoft). Briefly, parameters for "Select by Color Tool" in GIMP software were selected as follows: Antialiasing, OFF; Feather edges, OFF; Select transparent areas, OFF; Sample merged, OFF; Threshold, 30.0; Selt by: Saturation. Then, vacant area without cells in every well of the plate was selected for confluency analysis. Here, we selected the vacant area of the plate (Figure 4B) as a whole, but not for each independent well, in order to attain equal extraction conditions between the wells by eliminating the influence of subjectivity upon counting. Subsequently, the quantity of extracted area (number of pixels) were transferred to Excel software in accordance with the instructions of GIMP (Colors > Info > Histogram) to create a bar graph (Figure 4C).

Tumorigenesis in nude mice

All animal experiments were performed with the approval of the Animal Experiments Committee of Osaka University (permission number: BikenA-H24-17-0). SAS cells that expressed Dox-inducible Myc-vector or Myc-ELAS1 were injected into the left side of the tongue of BALB/c Slc-nu/nu female nude mice (5–6 weeks old; Japan SLC, Inc.). Each mouse was injected with 2×10^6 cells (Figure 5A and Figure S10A) or 1×10^6 cells (Figure S10C) prepared in 50 μl of DMEM. The body weight of each mouse was measured every 2 or 3 days. Dox (1.6 mg per mouse) was intraperitoneally injected about twice per week (on the days indicated by orange arrows in Figure 5). In some cases (Figure 5A and Figure S10A), each mouse was intraperitoneally injected with 0.1 mg of irinotecan prepared in 100 μl of saline once per week (on the days indicated by red arrows in Figure 5 and Figure S10). IHC images were obtained using the implanted tumors of nude mouse sacrificed at 14 days (this mouse was derived from an independent experiment) or sacrificed at 40 days after implantation.

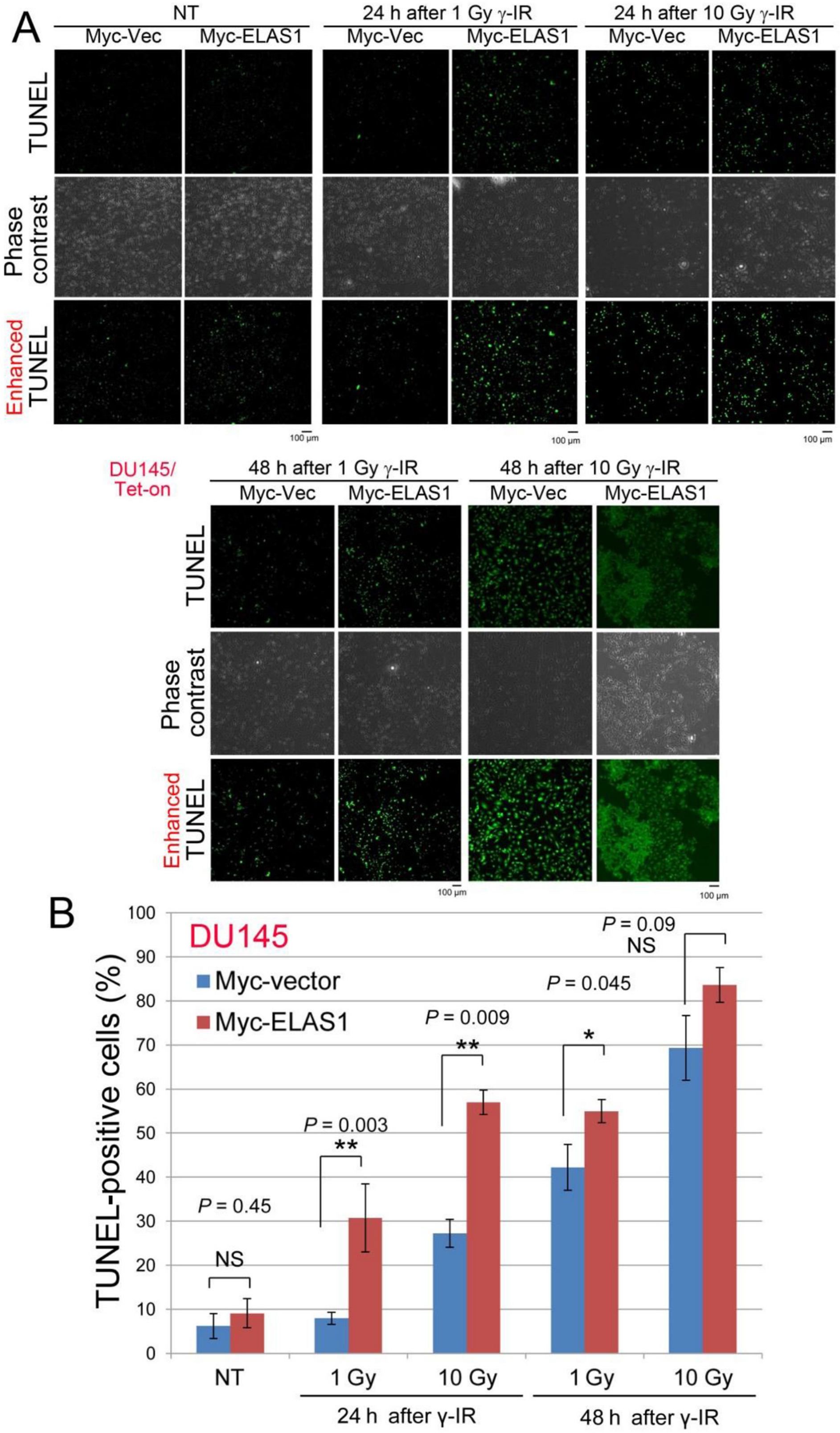

Supplementary Figure 1: ELAS1 induced apoptosis in DU145 cells after γ-IR treatment. **(A)** Typical microscope images of TUNEL-positive cells. **(B)** The bar graph shows the percentage of TUNEL-positive cells in the microscopic images. The data represents the means and S.D. of three independent measurements (200 cells were counted per measurement). NT, non-treated.

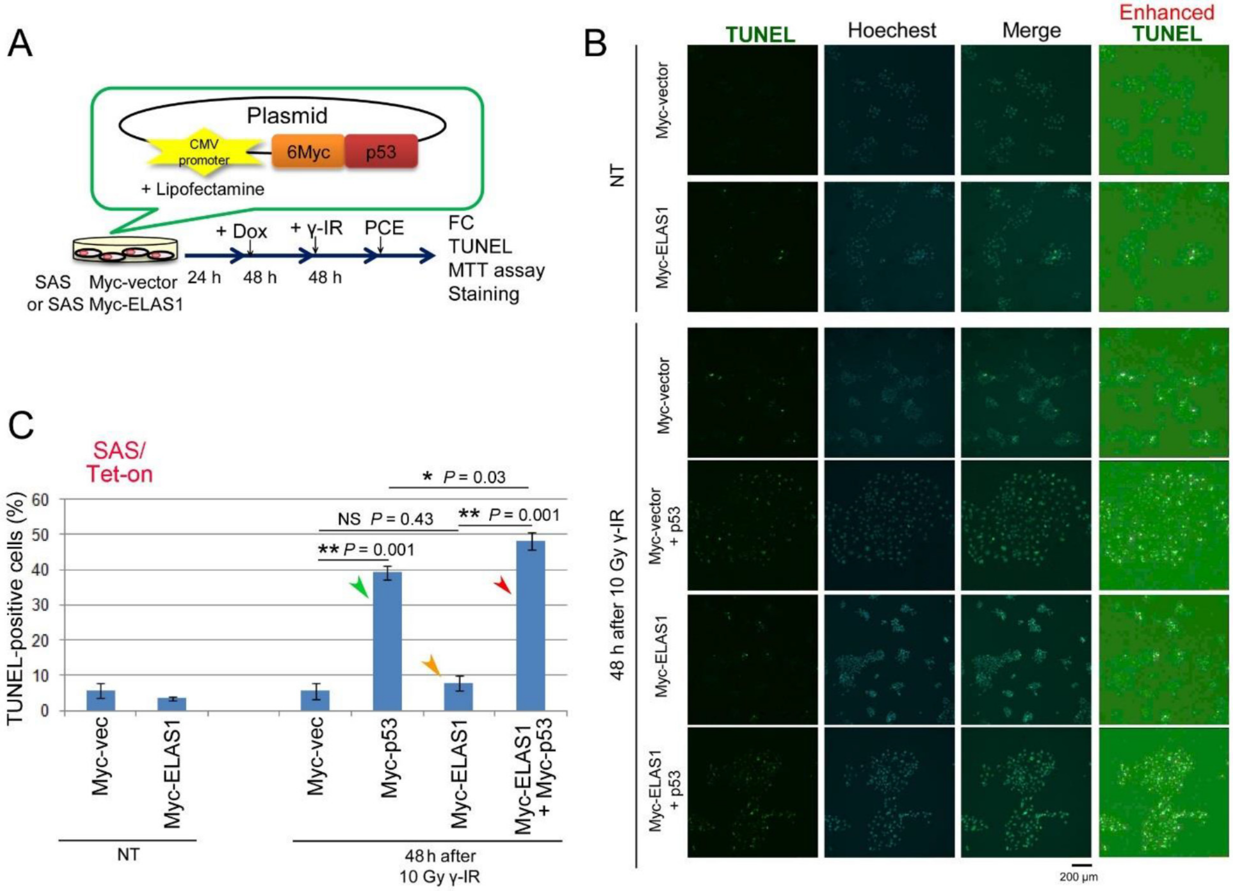

Supplementary Figure 2: TUNEL assays revealed ELAS1-mediated apoptosis in SAS cells after γ-IR treatment. **(A)** A schematic presentation of plasmid DNA that constitutively expressed 6Myc-tagged WT p53 (Myc-p53) proteins under the control of the CMV promoter when transfected into SAS/Tet-On Myc-ELAS1 cells using Lipofectamine. PCE, preparation of cell extract. This protocol was used for FC, TUNEL assay, MTT assay and crystal violet staining. **(B)** Typical microscope images of TUNEL-positive cells. DNA was also stained using Hoechst33258. **(C)** The bar graph shows the percentage of TUNEL-positive cells in the microscopic images. Green or red arrowheads indicate the bars for SAS/Tet-on cells expressing Myc-p53 proteins in the absence or presence of Dox-dependent expression of Myc-ELAS1 at 48 h after 10 Gy γ-IR treatments. The data represents the means and S.D. of three independent measurements (200 cells were counted per measurement). NT means non-treated cells used as a negative control.

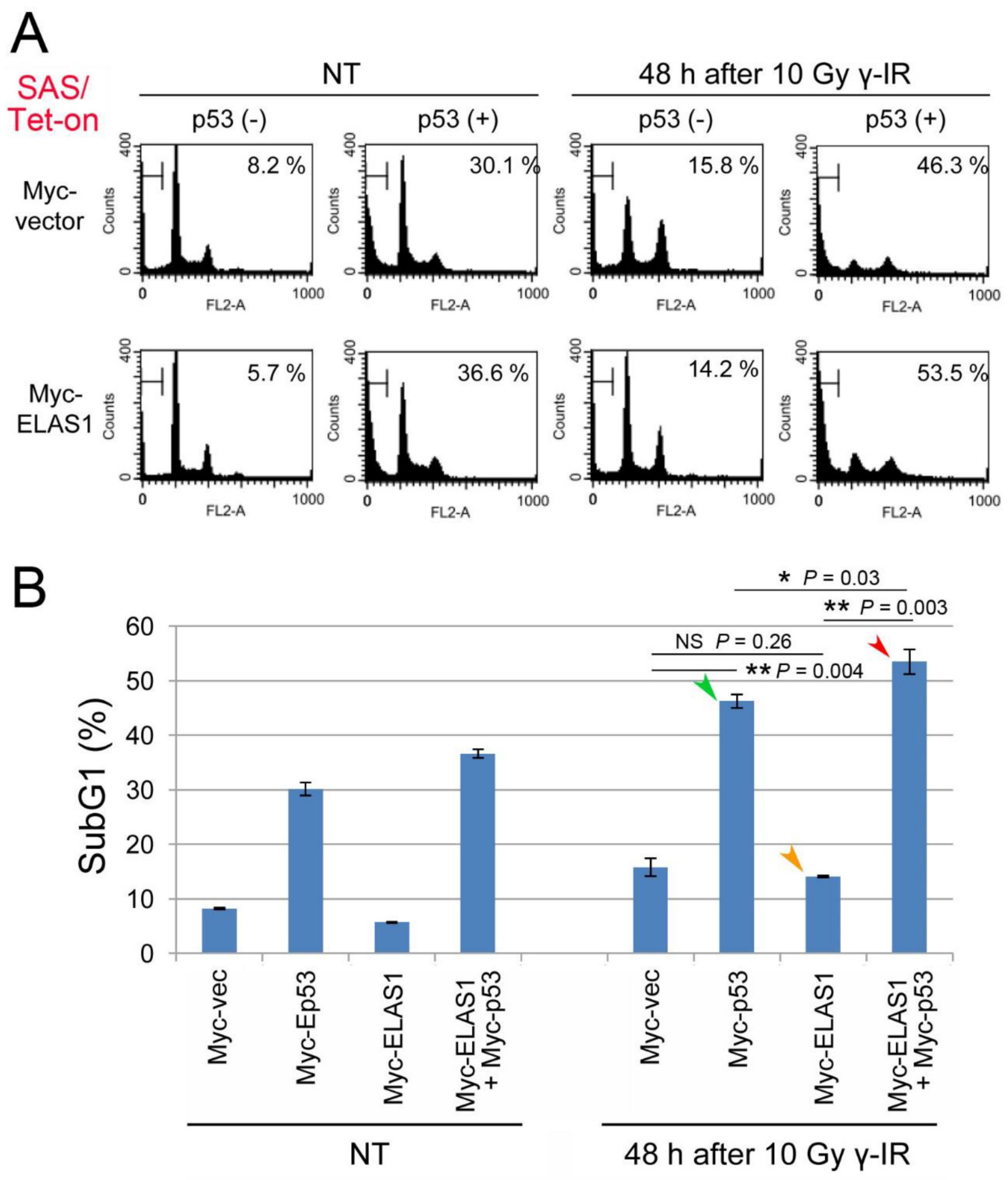

Supplementary Figure 3: FC analysis revealed ELAS1-mediated apoptosis in SAS cells after γ-IR treatment. (A) SAS/Tet-on cells expressing Myc (Myc-vector), Myc-p53, Myc-ELAS1, or Myc-p53 and Myc-ELAS1 proteins were subjected to FC analysis. Cells were stained with propidium iodide and the cell cycle profiles were determined by flow cytometry. Typical FC patterns are shown with percentages for the sub-G1 population cells. Data were obtained at 48 h after 10 Gy γ-IR treatments. NT means non-treated cells used as a negative control. Experimental protocol is shown in Supplementary Figure 2A. **(B)** The bar graph shows the percentage of cells in the sub-G1 population. Green or red arrowheads indicate the bars for SAS/Tet-on cells expressing Myc-p53 proteins in the absence or presence of Dox-dependent expression of Myc-ELAS1 at 48 h after 10 Gy γ-IR treatments. Data represent the mean and SD of three independent experiments (20,000 cells were counted per experiment).

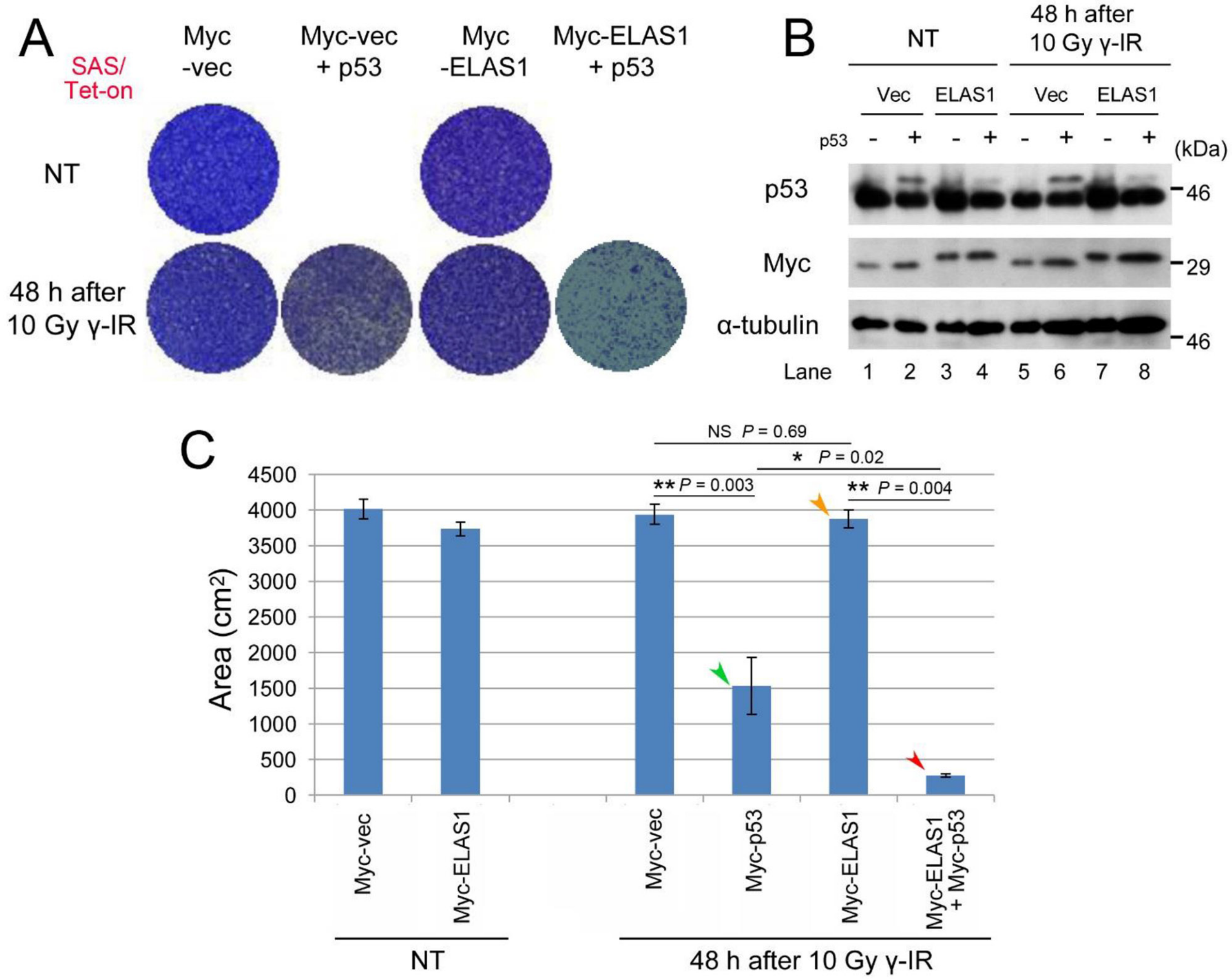

Supplementary Figure 4: Cell growth speed assay reveals efficient ELAS1-mediated apoptosis in SAS cells after γ-IR treatment. (A) SAS/Tet-on cells expressing Myc (Myc-vector), Myc-p53, Myc-ELAS1, or Myc-p53 plus Myc-ELAS1 proteins were subjected to the cell growth speed assay using crystal violet, which stains nuclei of ethanol-fixed cells, at 48 h after 10 Gy γ-IR treatment. NT signifies non-treated cells used as a negative control. The experimental protocol is shown in Figure 2E. **(B)** Wb examining expression of the indicated proteins. **(C)** The bar graphs show the area stained by crystal violet, which indicates the size of the area occupied by live cells. Green or red arrowheads indicate the bars for SAS/Tet-on cells expressing Myc-p53 proteins in the absence or presence of Dox-dependent expression of Myc-ELAS1 at 48 h after 10 Gy γ-IR treatments. Data represent the mean and SD of three independent measurements.

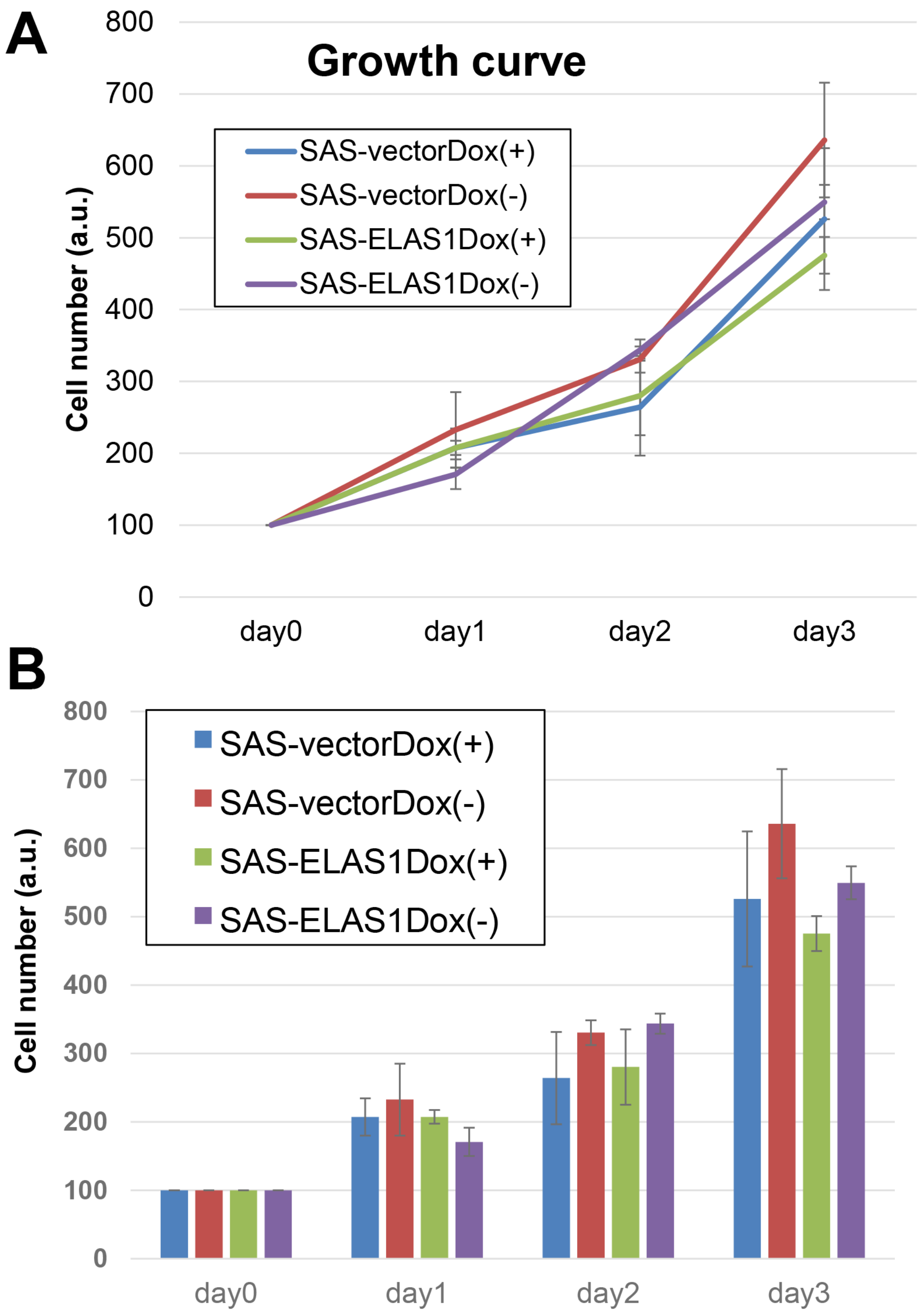

Supplementary Figure 5: Growth curve for SAS/Tet-On cells. **(A, B)** Line graph (A) and bar graph (B) presentation of cell growth profiles for SAS/Tet-On Myc-vector and SAS/Tet-On Myc-ELAS1 cells in the absence (-) or presence (+) of Dox.

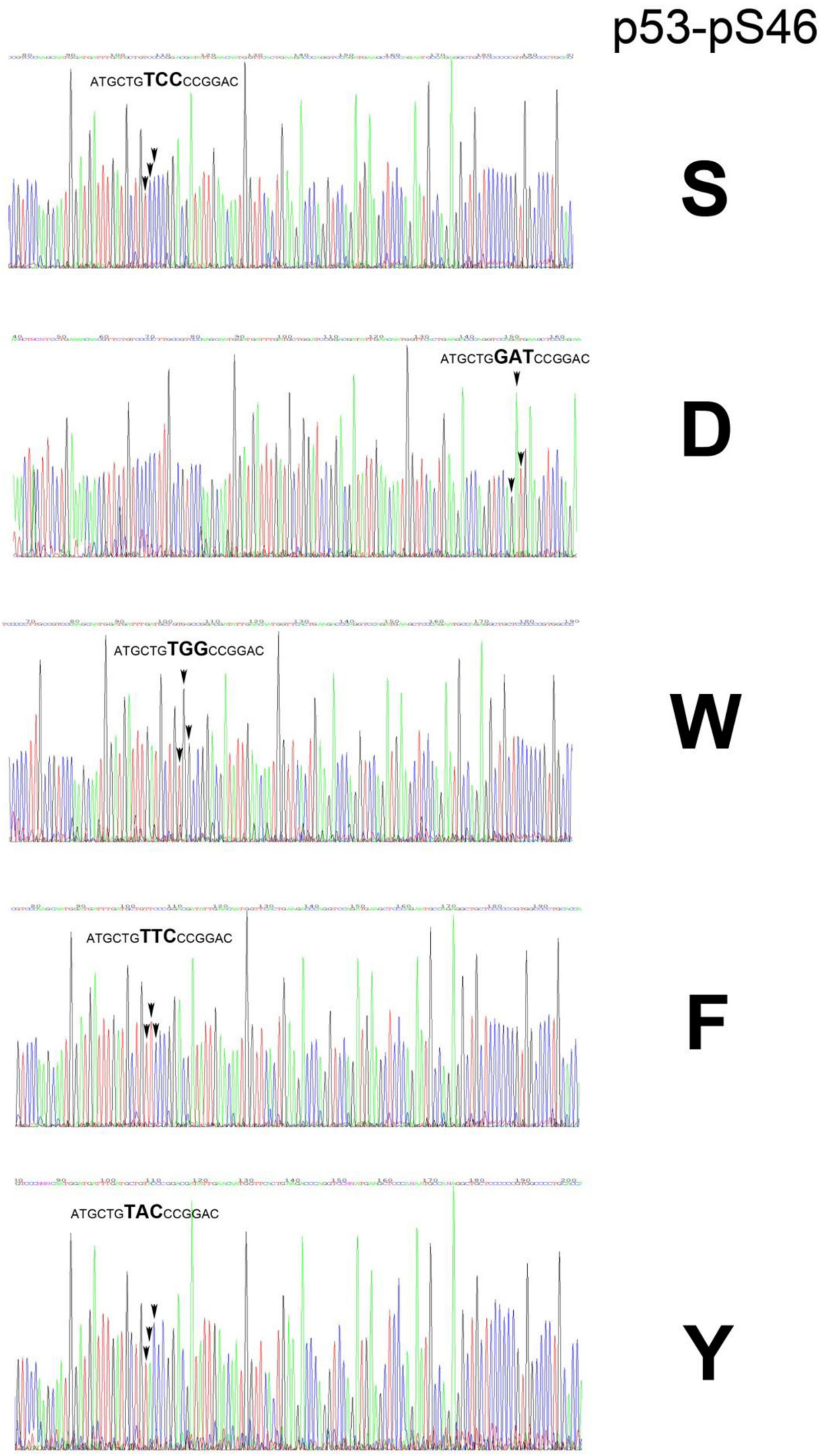

Supplementary Figure 6: Confirmation of nucleotide sequences at the mutation point of p53-S46. DNA sequencing ladder around p53-S46 is shown for each mutant.

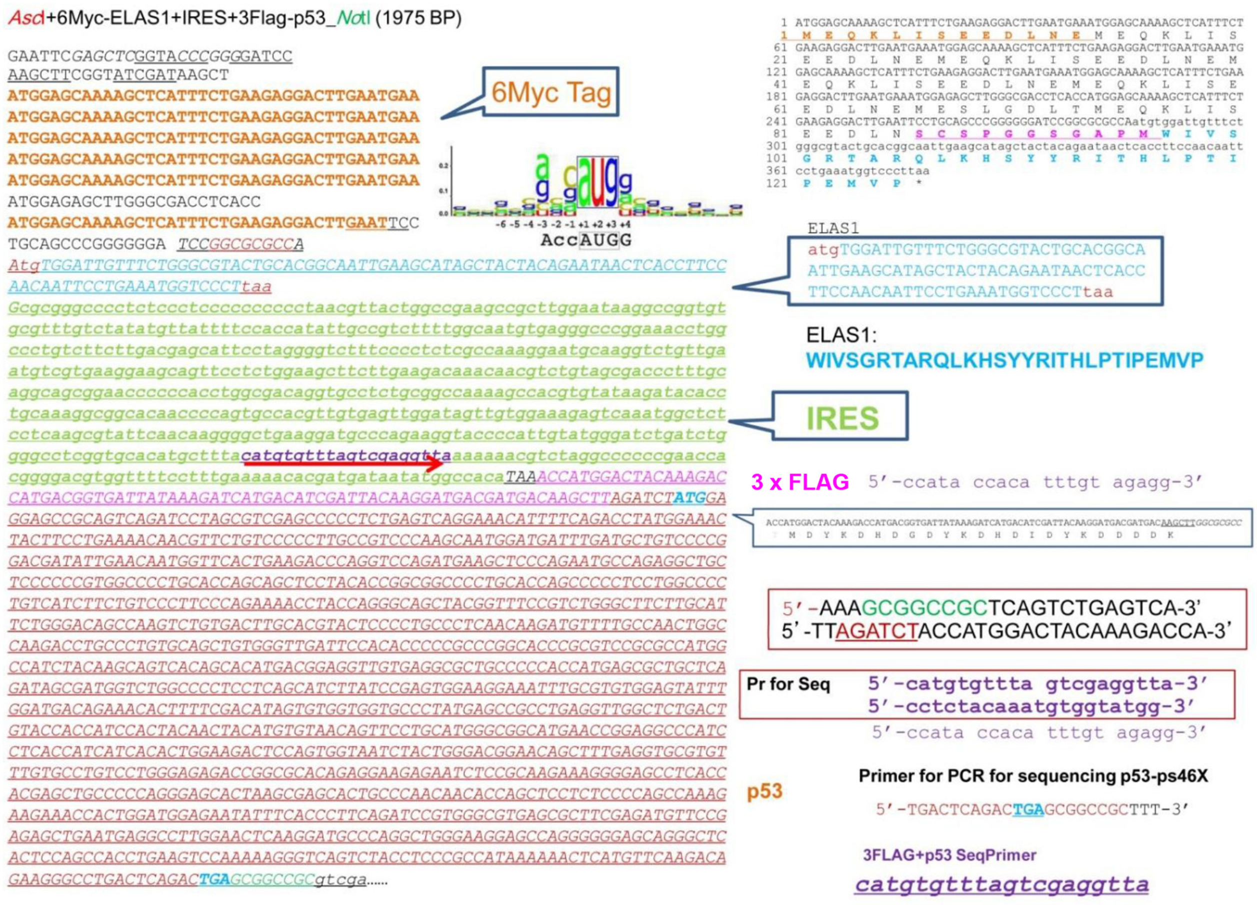

Supplementary Figure 7: Structure of Myc-ELAS1_IRES_FLAG-p53 construct. DNA sequence of Myc-ELAS1_IRES_F-p53 construct and relevant information are schematically presented.

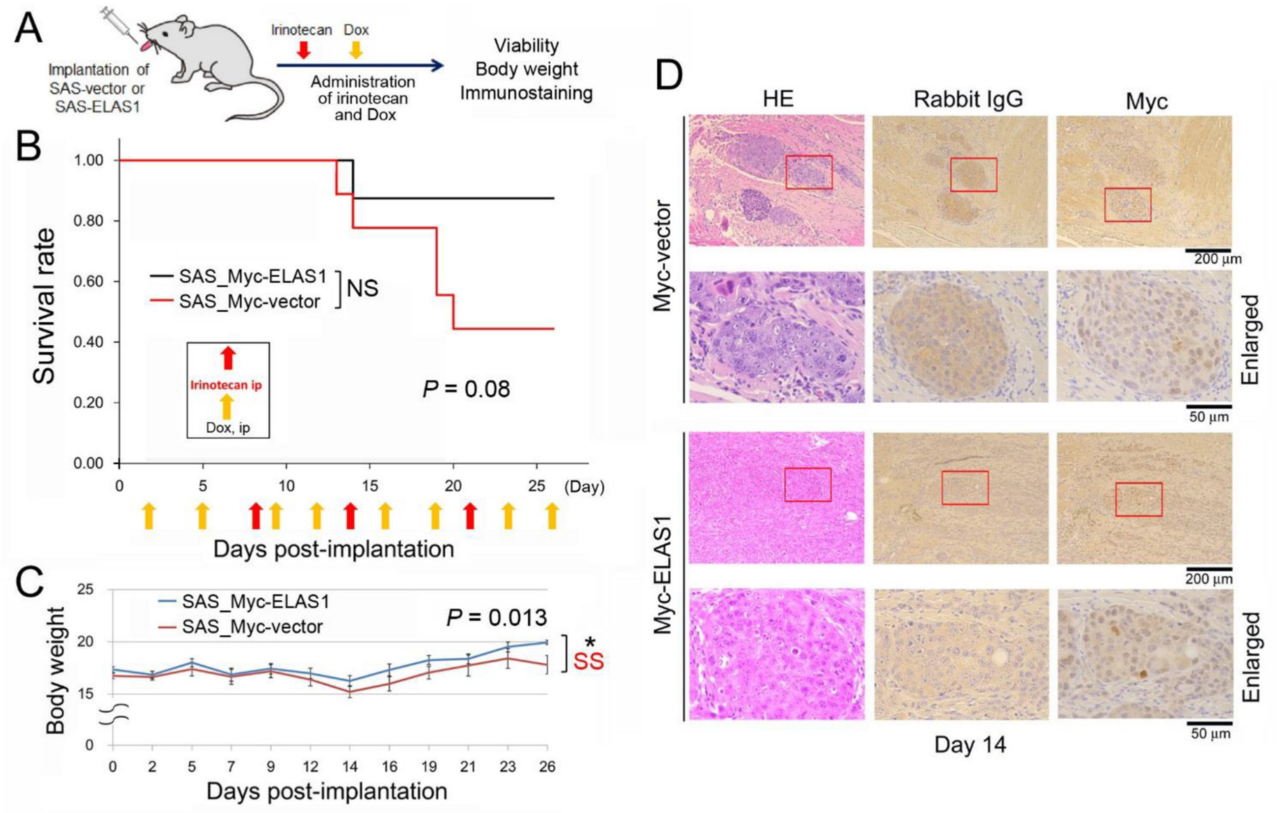

Supplementary Figure 8: Nude mice implanted with SAS/Tet-On Myc-ELAS1 cells in their tongue live longer than those implanted with SAS/Tet-On Myc-vector cells. **(A)** A schematic presentation of the experimental protocol. **(B)** Survival curves of mice implanted with SAS/Tet-On Myc-vector or SAS/Tet-On Myc-ELAS1 cells in their tongue. Dox (1.6 mg per mouse) was intraperitoneally injected about twice per week (on the days indicated by orange arrows). In addition, each mouse was also intraperitoneally injected with 0.1 mg of irinotecan prepared in 100 µl of saline once per week (on the days indicated by red arrows). **(C)** Body weight of each mouse was measured at indicated date. SS, statistically significant (P = 0.013). **(D)** Typical IHC images obtained using anti-Myc antibody confirm efficient expressions of Myc or Myc-ELAS1 proteins in Myc-vector or Myc-ELAS1 expressing SAS/Tet-On cells, respectively, in the implanted tumors of nude mouse sacrificed at 14 days after implantation; this mouse was derived from an independent experiment. Enlarged images of the region encircled by red rectangles are also shown.

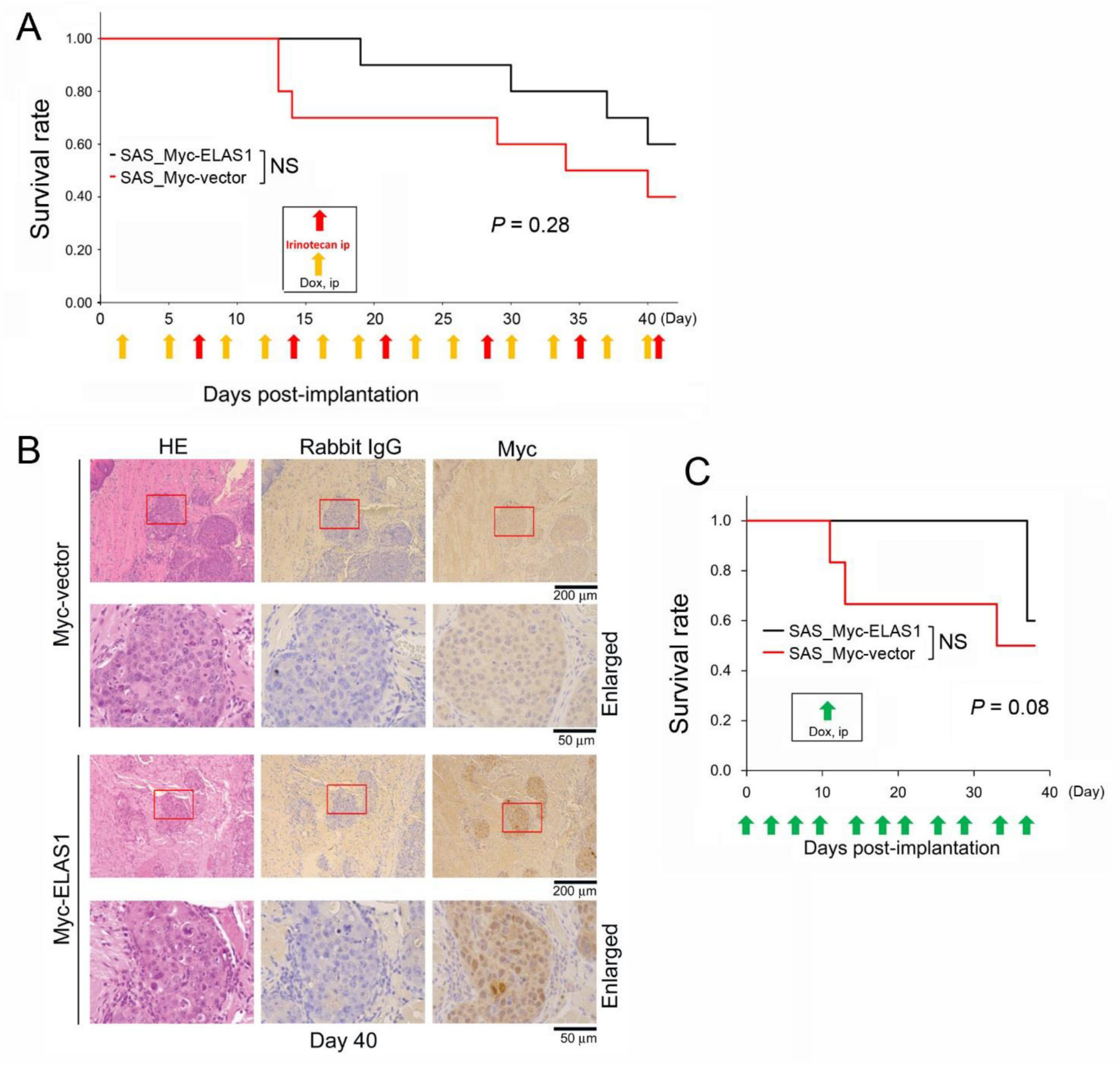

Supplementary Figure 9: Survival curves of mice implanted with SAS/Tet-On Myc-vector or SAS/Tet-On Myc-ELAS1 cells in their tongue. **(A)** Survival curves of mice implanted with SAS/Tet-On Myc-vector or SAS/Tet-On Myc-ELAS1 cells in their tongue. Dox (1.6 mg per mouse) was intraperitoneally injected about twice per week (on the days indicated by orange arrows). In addition, each mouse was also intraperitoneally injected with 0.1 mg of irinotecan prepared in 100 µl of saline once per week (on the days indicated by red arrows). **(B)** Typical IHC images obtained using anti-Myc antibody confirm efficient expressions of Myc or Myc-ELAS1 proteins in Myc-vector or Myc-ELAS1 expressing SAS/Tet-On cells, respectively, in the implanted tumors of nude mouse after 40 days of implantation. **(C)** In this experiment, only Dox (1.6 mg per mouse) was intraperitoneally injected about twice per week (on the days indicated by green arrows).

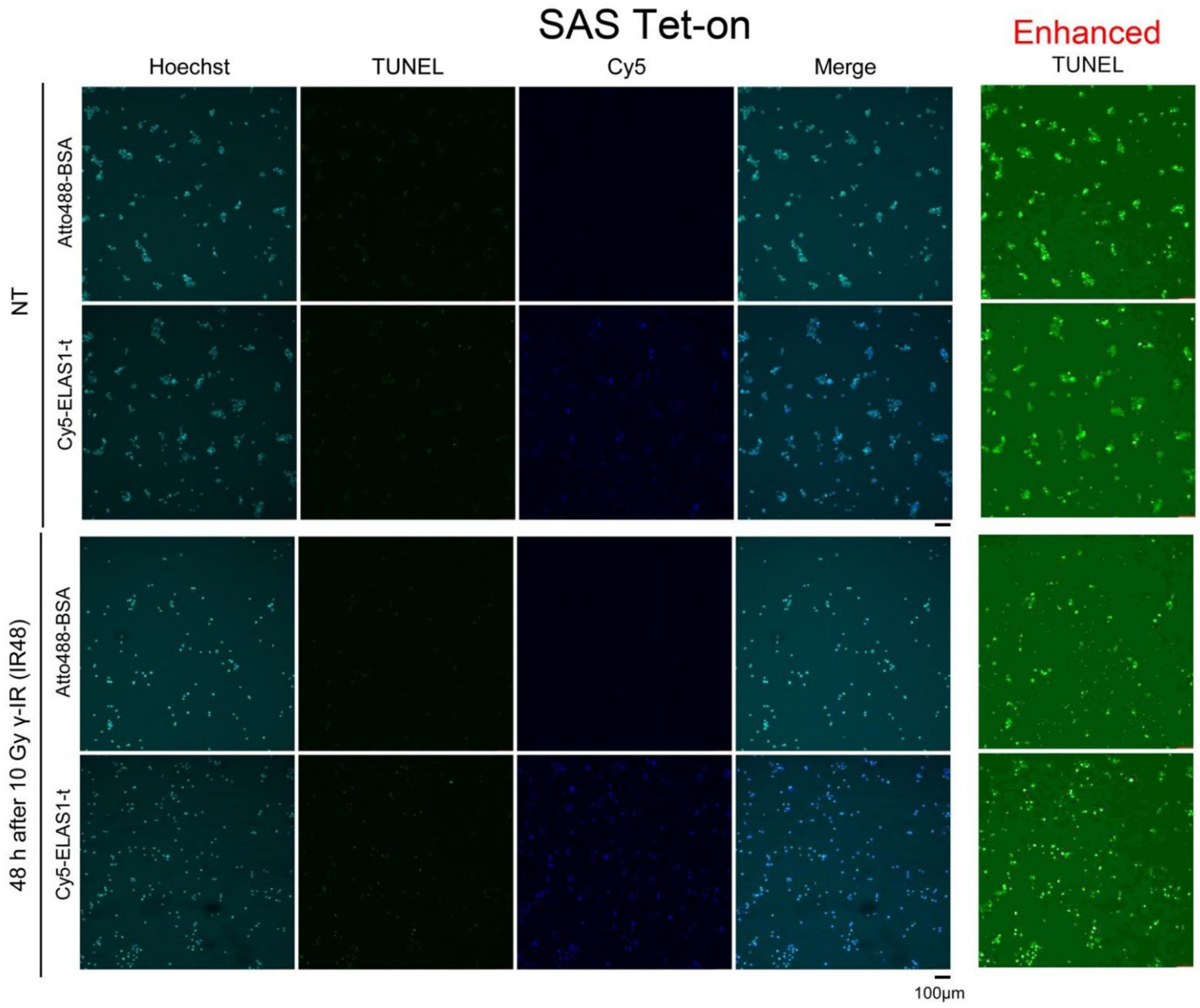

Supplementary Figure 10: Typical microscope images of TUNEL-positive cells. SAS Tet-on cells harboring Cy5C_ELAS1-t cells were subjected to TUNEL assays at 48 h after 10 Gy IR (IR48) or without IR (NT, non-treated). DNA was visualized by Hoechst33258 staining. Atto488-BSA was used as a negative control. Rightmost panels show the contrast-enhanced TUNEL images. Bar, 0.1 mm.
